# Precise Sequence Control and Thermal Characterization of *N*‑Substituted Maleimide Copolymers Containing Hydrazine Structure

**DOI:** 10.1021/polymscitech.6c00081

**Published:** 2026-07-06

**Authors:** Akikazu Matsumoto, Natsuki Okada, Yuki Nakai, Tomohiro Suzuki, Yasuhito Suzuki

**Affiliations:** † Department of Applied Chemistry, Graduate School of Engineering, 12936 Osaka Metropolitan University, 1-1, Gakuen-cho, Naka-ku, Sakai, Osaka 599-8531, Japan; ‡ Department of Applied Chemistry, Faculty of Engineering, Osaka Prefecture University, 1-1, Gakuen-cho, Naka-ku, Sakai, Osaka 599-8531, Japan

**Keywords:** *N*-aminomaleimide, degradable polymer, exothermic decomposition, glass transition temperature, hydrazine, inhibitor, isomerization, monomer reactivity ratio, monomer synthesis, penultimate unit effect, radical copolymerization, sequence control, terminal model, thermal stability

## Abstract

*N*-Substituted maleimides are highly reactive and have been used for the synthesis of various thermoresistant polymer materials by radical copolymerization with vinyl monomers. In this study, we synthesized several maleimide monomers with *N*-amino substituents, i.e., *N-*(*N′,N′*-dimethylamino)­maleimide, *N-*(*N′,N′*-diphenylamino)­maleimide, and *N*-morpholinomaleimide, using *N,N*-disubstituted hydrazine compounds and maleic anhydride as the starting materials. We carried out radical copolymerization of the *N*-aminosubstituted maleimides with styrene, methyl acrylate, isobutyl vinyl ether, and limonene, and analyzed the composition and arrangement of the repeating monomer units in the produced copolymers in order to elucidate the detailed copolymerization mechanism, including propagation reactions controlled by terminal and penultimate unit models. In the copolymerization of the maleimides with styrene and methyl acrylate, alternating and random copolymers were formed, respectively, by the propagation processes according to the terminal model. In the copolymerization with isobutyl vinyl ether and limonene, copolymers containing predominant AAB repeating structures were formed under the penultimate unit control. It was also clarified that the introduction of compact and electron-donating *N*-substituents is promising for the induction of the penultimate unit effect. Then, the chemical and physical properties of the copolymers were characterized. The 10% weight-loss temperatures of the copolymers were in the range of 300–350°C in thermogravimetric analysis, indicating their excellent thermal stability. It was found that the copolymers containing a large number of maleimide units decomposed exothermically regardless of the atmosphere because the copolymers contained an N–N bond in their side chain, similarly to other nitrogen-rich polymer materials containing N–N bonds. The glass transition temperatures of the copolymers varied in the range of 40–220°C depending on the kind of vinyl monomers used and the composition of the copolymers, which agreed well with the values predicted from Fox’s equation. We also attempted to synthesize *N*-(*N′*-phenylamino)­maleimide derived from a mono-substituted hydrazine compound and evaluated the role of the isolated 6-membered cyclic product on radical polymerization reactions.

## Graphical abstract

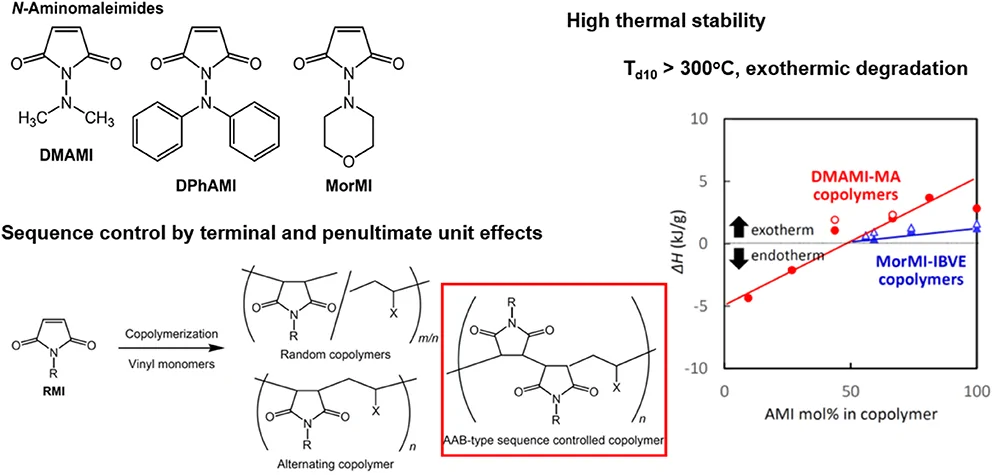

## Introduction

*N*-Substituted maleimides (RMIs), which contain highly reactive unsaturated double bonds, are frequently used in chain polymerization and copolymerization by radical or anionic mechanism^1–3^ as well as in various step-wise polymerizations^3–6^ (Scheme 1(a)–(d)). RMI structures can be used not only as starting monomers for polymer synthesis but also as reactive functional groups in the side chain or at the chain end of polymers using Diels–Alder addition and ene–thiol reaction.^3–8^ In fact, numerous polymer materials containing maleimide units have been developed, including sequence-controlled polymers,^9–12^ self-healing polymers,^13–16^ optical polymers,^17–19^ hydrogels,^20^ and bioconjugates.^21–23^ Thus, while maleimide structures are useful in polymer synthesis through various reaction processes, it should be noted that one of the most common applications of the RMIs is simply radical copolymerization. When the RMIs are copolymerized with various types of vinyl monomers, they exhibit a variety of properties depending on the monomer used in the copolymerization, as follows.

**Scheme 1:**
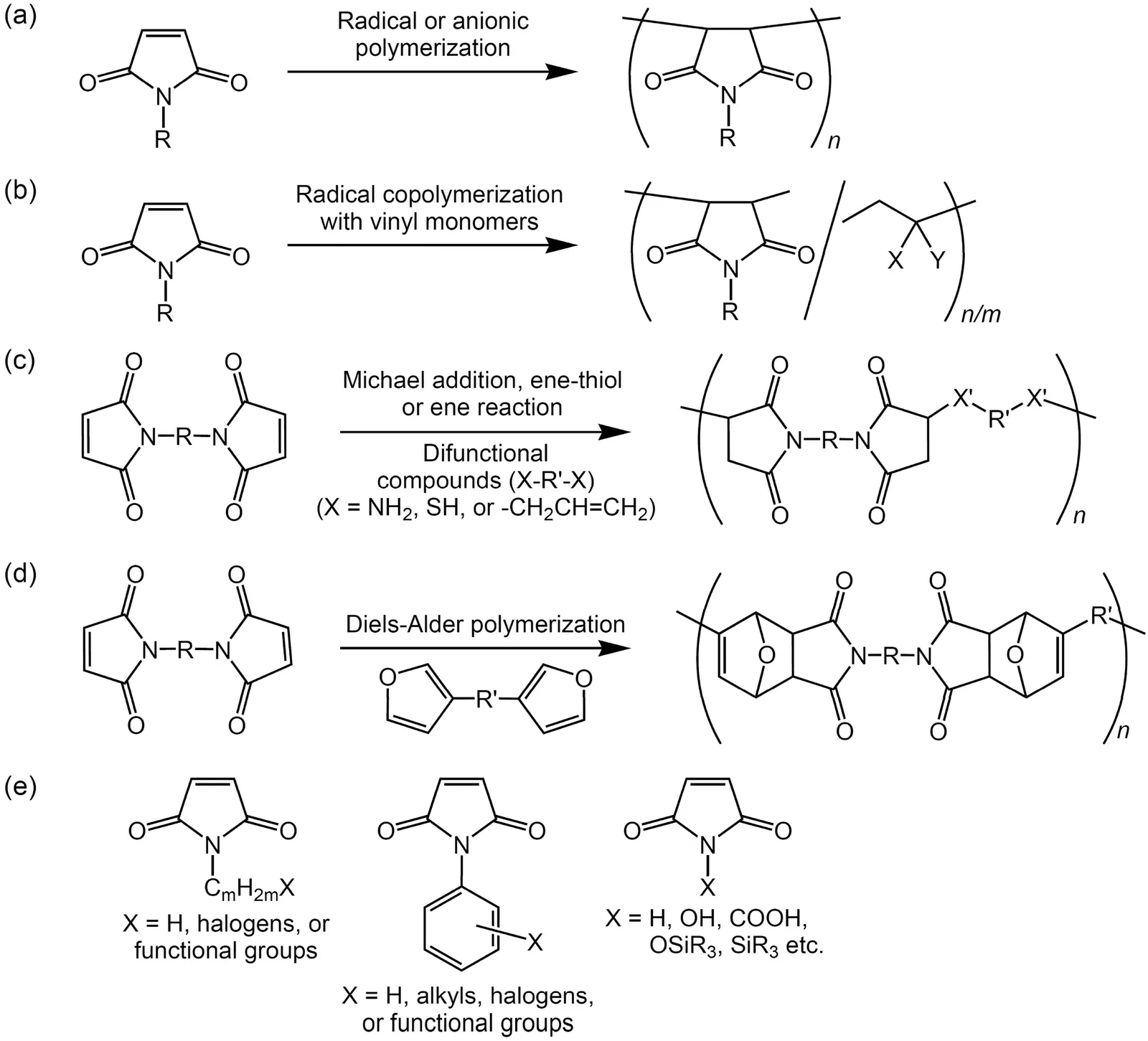
Typical Reaction Processes for Polymer Synthesis Using the RMIs^a^

a (a) Radical or anionic polymerization, (b) radical copolymerization with vinyl monomers, (c) addition polymerization of bismaleimides with difunctional compounds by Michael addition, ene–thiol reaction, and ene reaction, (d) Diels–Alder polymerization, and (e) chemical structure of *N*-substituted maleimides with various *N*-substituents.

Typical sequence structures of the copolymers produced by radical copolymerization of the RMIs with vinyl monomers are depicted in Scheme 2. RMIs are copolymerized with electron-donating monomers such as styrene (St) and vinyl ethers in the presence of a radical initiator to give high-molecular-weight alternating copolymers.^24–28^ On the other hand, when RMI is combined with electron-withdrawing monomers such as acrylates and methacrylates, random copolymers are formed.^25,29–31^ The copolymer composition can be easily adjusted according to the feed composition during random copolymerization, and therefore, physical properties such as glass transition temperatures (*T*_g_) are precisely controlled. In addition, copolymerization of the RMIs with nonconjugated monomers such as vinyl chloride, vinyl acetate, and olefins is possible.^29,32–36^ In the copolymerization of the RMIs and olefins, copolymers with different copolymer compositions and sequence distributions are produced according to the chemical structures of olefins. For example, the copolymerization of *N*-methylmaleimide (MMI) with isobutene yields highly alternating copolymers,^37,38^ with high thermal stability (onset temperature over 350°C and *T*_g_ over 150°C), excellent optical properties (visible light transmittance over 95%), and mechanical properties (bending strength more than 130 MPa and bending modulus more than 4.5 GPa). This kind of copolymer has been developed as one of the important candidates for heat-resistant and transparent polymer materials for industrial glass substitutes.^38^ The excellent thermal stability, i.e., high *T*_g_ and thermal decomposition onset temperature, of the maleimide copolymers is due to the chemically stable imide ring structure with restricted free rotation around the main chain.

**Scheme 2:**
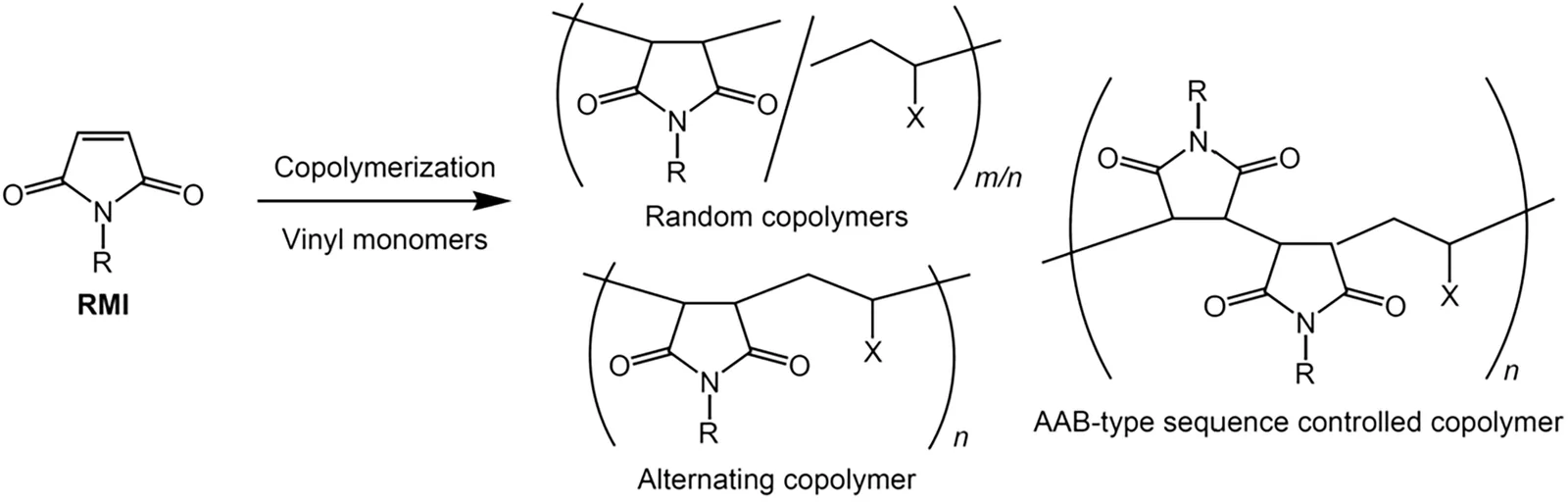
Repeating Structure of Random, Alternating, and Periodic (AAB-type) Copolymers Synthesized by Radical Copolymerization of the RMIs with Vinyl and Olefin Monomers

On the other hand, copolymerization of the RMIs with bulky and rigid nonconjugated olefins, such as limonene (Lim),^36,39^ diisobutene,^34–36^ β-pinene,^40^ valencene,^41^ and other olefins,^42,43^ induces the penultimate unit effect, resulting in the formation of copolymers containing preferential AAB repeating units. The effect of the chemical structure of the *N*-substituent of RMI on the sequence control has been investigated. Electron-withdrawing *N*-substituents tend to induce alternating copolymerization, whereas electron-donating *N*-substituents tend to induce AAB-type controlled copolymerization.^36^ It has been pointed out that increasing the steric hindrance of the *N*-substituent and decreasing the rate of homopropagation of the maleimide unit lead to the formation of alternating copolymers rather than the AAB-type control.

The most typical and convenient method for synthesizing RMI is the intramolecular cyclocondensation of the corresponding *N*-substituted maleamic acid, which is formed by the reaction of maleic anhydride with amines. Dehydration with acetic anhydride in the presence of a base catalyst^1^ or dehydration by heating in the presence or absence of an acid catalyst^44^ is used for condensation. In order to realize highly functional and high-performance polymer materials, a large number of RMIs containing a characteristic functional group have been synthesized. However, most of the RMIs used so far have been prepared by introducing a functional group at a part of the substituent of *N*-phenyl- or *N*-alkylmaleimides (Scheme 1e). On the other hand, there are few reports on the polymerization or copolymerization of the RMIs containing a functional group directly on the nitrogen atom of the maleimide ring without an aromatic ring or an alkyl spacer.^45–48^ Here, a hydrazine derivative can be used as an amine to synthesize RMI containing an amino group as the *N*-substituent.^49–52^ Hydrazine derivatives are highly reactive and reductive compounds and used as liquid rocket fuel or propellant and intermediates for pharmaceuticals, agrochemicals, and industrial chemicals.^53–55^ However, reports on the polymerization of *N*-(substituted amino)­maleimides (AMIs) are extremely rare,^56,57^ and the details of the polymerization reactivity and the properties of the resulting polymers are still unknown.

In this study, we synthesized three kinds of AMI, i.e., *N-(N*′,*N*′-dimethylamino)­maleimide (DMAMI), *N-(N′,N′*-diphenylamino)­maleimide (DPhAMI), and *N*-morpholino-maleimide (MorMI) as shown in Figure 1, using the corresponding *N,N*-disubstituted hydrazine compounds according to a previously reported procedure,^49^ and then investigated the radical copolymerization characteristic of the AMIs. St, methyl acrylate (MA), isobutyl vinyl ether (IBVE), and Lim were selected as comonomers, and the composition and sequence structure of the resulting copolymers were analyzed in detail. We determined decomposition temperatures and enthalpies as well as *T*_g_ in order to clarify the thermal properties and decomposition mechanism of the copolymers containing a hydrazine structure in the side chain. We also attempted to synthesize *N*-(*N′*-phenylamino)­maleimide (PhAMI) derived from a mono-substituted hydrazine,^50–52^ and isolated a compound with an unexpected 6-membered ring structure. The polymerization ability of this cyclic product was evaluated, and it was found that it is available as a strong polymerization inhibitor rather than a monomer.

**Figure 1:**
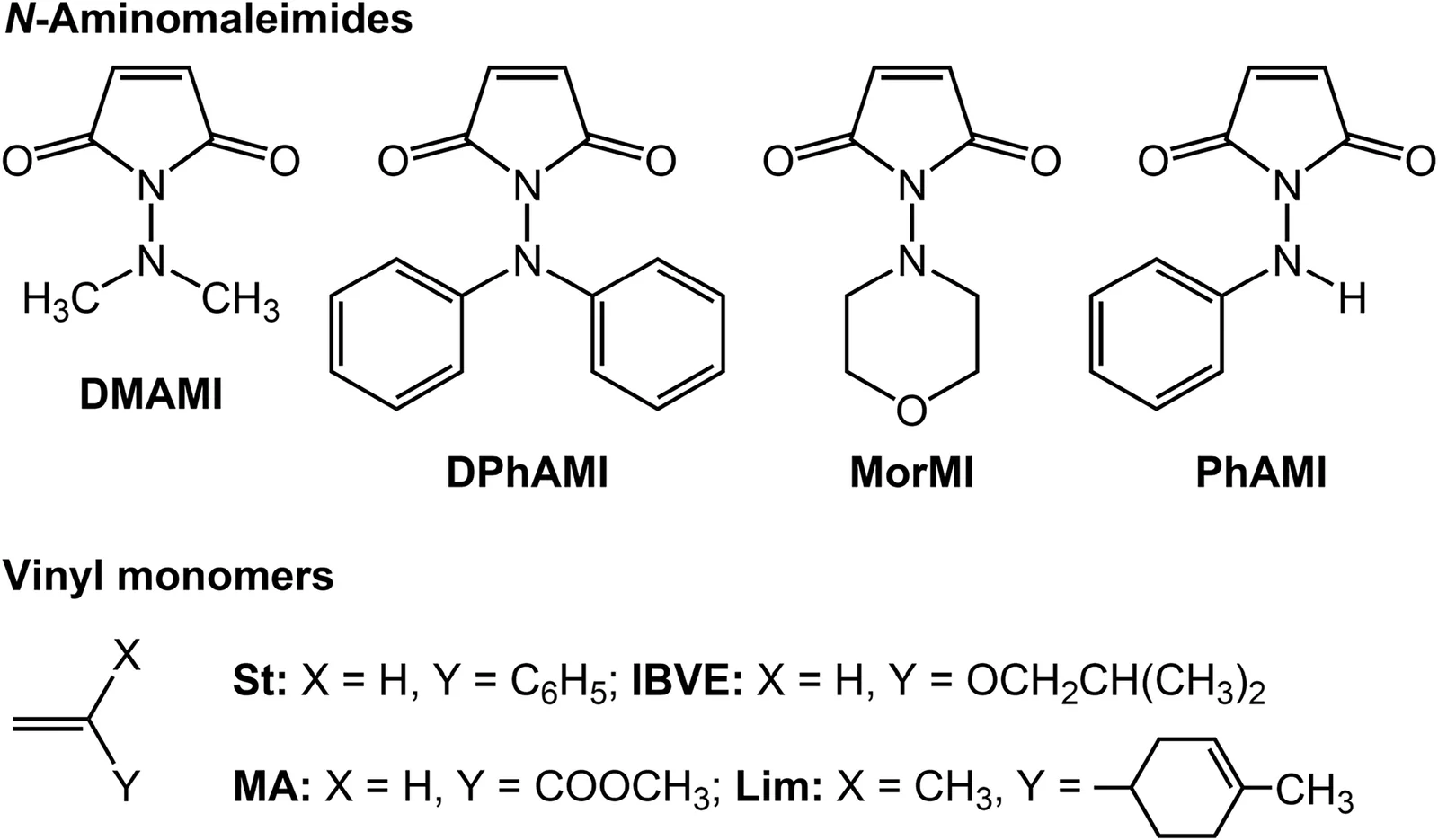
Chemical structure of *N*-aminomaleimides (AMIs) and vinyl monomers used in this study.

## Results and Discussion

### Copolymer Synthesis

Radical copolymerization of DMAMI, DPhAMI, and MorMI as the M_1_ monomers with St, MA, IBVE, and Lim as the M_2_ monomers was carried out in chloroform at 60°C using AIBN as a radical initiator at different monomer ratios in a range of [M_1_]/[M_2_] = 1/9 to 9/1 (mol/mol). The produced copolymers were purified by repeated reprecipitation using chloroform and methanol as solvent and nonsolvent, respectively. The results of the copolymerization are shown in Table 1. The number-average molecular weight (*M*_n_) of the produced copolymers was several tens of thousands or more. With an increase in the AMI content in the feed, *M*_n_ decreased in most cases. Homopolymerization of the AMIs was possible. Because the reactivity of the AMIs was not high, a longer polymerization time (9–40 h) was required to obtain a polymer in a moderate yield (12–36%). The polymerization rate and the *M*_n_ value were in the order of DMAMI > MorMI > DPhAMI. It was in good agreement with the magnitude of the steric hindrance of the *N*-substituents, as will be discussed later. The most isolated copolymers were colorless powder. The copolymers, including high AMI contents and the AMI homopolymers, were pale yellow. All the polymers were soluble in chloroform, THF, and toluene, and insoluble in methanol, ethanol, and *n*-hexane. The AMI homopolymers were also soluble in acetone and dimethyl sulfoxide.

**Table 1:** Radical Copolymerization of AMI (M_1_) with Vinyl Monomers (M_2_) with AIBN in Chloroform at 60 °C^a^

M_1_	M_2_	M_1_ mol % in comonomer	time (h)	yield (%)	M_1_ mol % in copolymer^b^	*M*_n_ × 10^–4^	*M*_w_/*M*_n_
DMAMI	St	10.0	1.2	18.8	40.7	4.3	2.0
		20.0	1.0	19.6	46.5	6.8	2.0
		30.0	0.5	21.0	47.7	12.6	2.6
		40.0	0.5	12.5	47.6	16.0	3.0
		50.0	0.5	27.8	50.3 [52.4]	7.4	2.4
		60.0	0.7	22.8	49.6	7.0	2.5
		70.0	1.0	32.6	50.4	17.0	1.9
		80.0	0.5	29.1	53.6	10.9	3.1
		90.0	1.0	14.0	56.4	6.9	2.9
		100	9	35.8	100	1.2	2.0
DMAMI	MA	10.0	4	32.3	9.5	2.7	2.2
		30.0	4	38.9	26.8	2.4	2.3
		50.0	10	30.3	43.8 [42.0]	1.1	2.1
		70.0	8	30.1	66.7	1.6	2.2
		90.0	8	22.3	81.2	1.0	2.0
DMAMI	IBVE	10.0	1.5	18.6	56.5	1.6	2.0
		30.0	0.4	23.4	58.1	3.3	2.4
		50.0	0.4	11.8	60.7	3.7	2.6
		70.0	1.0	39.3	66.4	2.3	4.6
		90.0	1.5	17.2	74.6	1.7	4.0
		95.0	2.5	4.9	90.6	0.68	2.8
DMAMI	Lim	20.0	10	3.2	60.8	0.51	2.0
		33.3	9	7.6	65.3	0.53	2.0
		50.0	8	17.5	64.0	0.94	1.5
		66.7	9	10.1	65.7	0.81	2.2
		80.0	10	35.4	66.6	0.45	1.8
MorMI	IBVE	10.0	3.0	14.4	56.0	5.0	2.0
		20.0	1.0	12.8	54.9	3.9	1.7
		50.0	4.0	20.0	59.3	1.4	3.2
		80.0	2.3	9.2	68.8	1.1	1.8
		90.0	4.0	1.9	74.0	0.78	1.7
		100	20	20.0	100	0.30	1.8
DPhAMI	IBVE	20.0	2	1.0	51.4	3.0	1.9
		50.0	6	2.0	56.5	2.6	3.1
		80.0	2	2.0	55.5	0.52	3.4
		90.0	15	8.5	66.2	0.93	1.5
		100	40	12.0	100	0.15	2.1

a Copolymerization conditions: [M_1_] + [M_2_] = 1.0 mol/L and [AIBN] = 20 mmol/L.

b Values in parentheses indicate the data determined based on the monomer consumption rates.

The composition of the copolymers was determined from the integral ratio of the characteristic peaks in their ^1^H NMR spectra (see the Supporting Information). For some copolymerization systems, CDCl_3_ was used as a solvent, and the consumption rates of vinyl and maleimide groups were directly determined by ^1^H NMR measurements of a copolymerization solution. The ratios of the monomer consumption rates were consistent with the copolymer composition determined by structural analysis of the produced copolymers (Table 1).

The copolymerization of DMAMI with St gave highly alternating copolymers, and the content of DMAMI in the copolymers was within a narrow range of 40.7–56.4 mol %, independent of the feed monomer composition (10.0–90.0 mol %). The copolymerization proceeded rapidly, and a high-molecular-weight copolymer (*M*_n_ = 4.3–17.0 × 10^4^) was formed. In contrast, the copolymerization with MA formed a random copolymer, the composition of which significantly depended on the feed composition. The rate of copolymerization with MA was relatively low, and several hours were required to obtain copolymers with yields of several 10% or more.

The copolymerization with IBVE resulted in the formation of copolymers with DMAMI contents of 56.5–90.6 mol %, indicating that DMAMI was preferentially incorporated into the copolymers. The copolymerization with Lim similarly gave copolymers containing a large and almost constant amount of DMAMI units (60.8–66.6 mol %), regardless of the feed composition. The copolymerization of DPhAMI and MorMI with IBVE proceeded similarly, resulting in the formation of the copolymers containing a large amount of AMI units. It was clarified that the type of substituent on the *N*-amino group dominated the rate of polymerization and the composition of the copolymers. The rate of copolymerization and the content of AMI incorporated in the copolymers decreased in the order of DMAMI > MorMI > DPhAMI, being similar to the results for homopolymerization. As will be described later, the copolymerization of DMAMI with IBVE or Lim is controlled by the penultimate unit effect. It has been reported that the copolymerization rate (or polymer yield) and the molecular weight of the resulting copolymers tend to decrease when the penultimate unit effect is observed during copolymerization. It is due to a change in the balance of the homo- and cross-propagation rates for controlling the composition and sequence structures of the copolymers.^34,39,58^

### Monomer Reactivity Ratios and Propagation Mechanism

The composition curves of copolymerization of the AMIs were analyzed by using the terminal and penultimate unit models, and the results are shown in Figure 2 and Table 2. The experimental values obtained from copolymerization with St agreed well with the calculated curves based on the terminal model (Figure 2a**)**. The monomer reactivity ratios obtained by the nonlinear least-squares method^34,58^ were *r*_1_ = 0.034 and *r*_2_ = 0.050. Both of these values were close to 0, indicating a highly controlled alternating copolymerization. The copolymerization of MA could also be explained by a terminal model. The monomer reactivity ratio (*r*_1_ = 0.69, *r*_2_ = 1.11) was relatively close to unity (Figure 2b). This indicates that MA had slightly higher reactivity than DMAMI in the random copolymerization.

**Figure 2:**
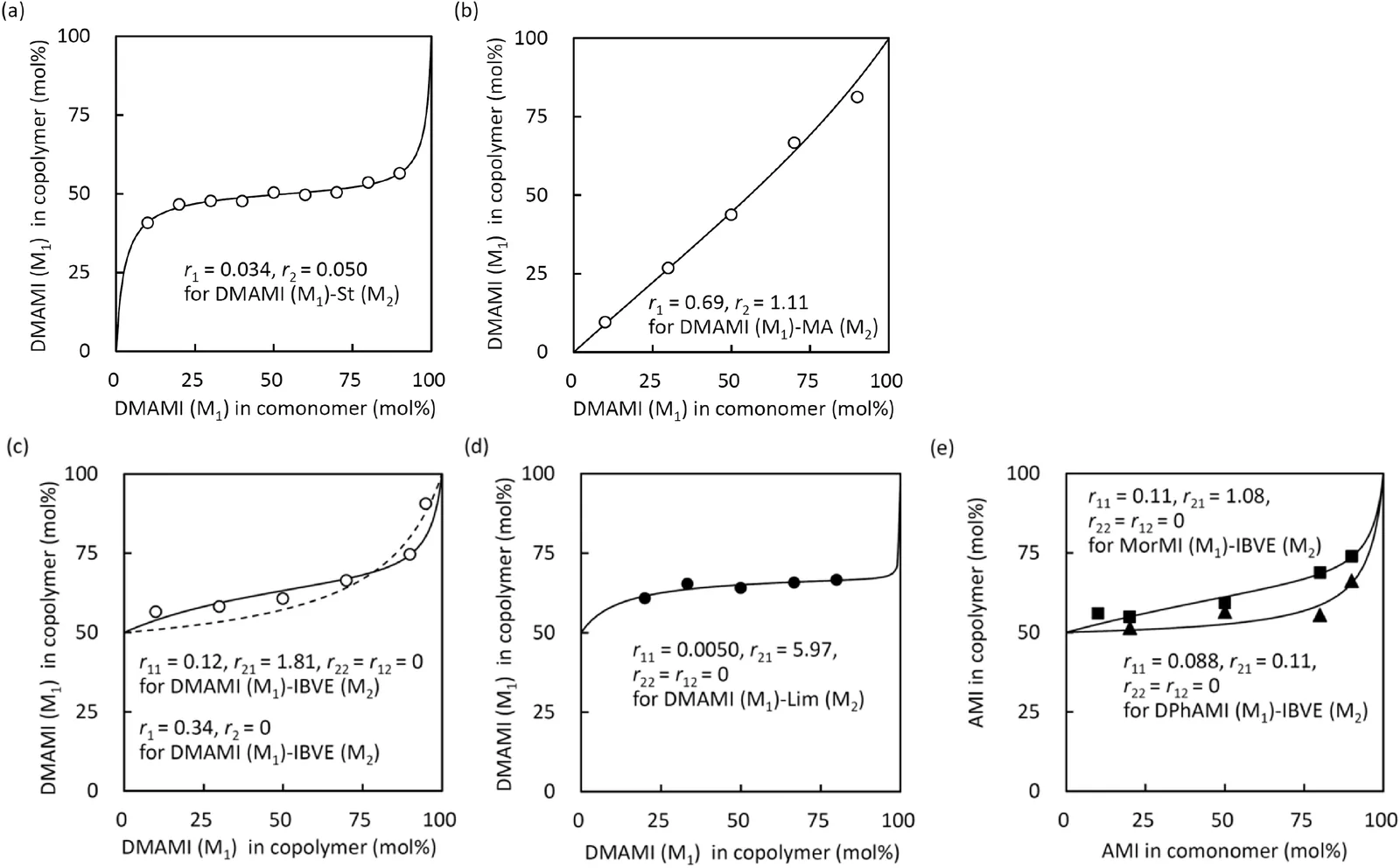
Comonomer-copolymer composition curves for radical copolymerization of (a) DMAMI (M_1_) with St (M_2_), (b) DMAMI (M_1_) with MA (M_2_) using the terminal model, (c) DMAMI (M_1_) with IBVE (M_2_) using the terminal model (broken curve) and the penultimate unit model (solid curve), (d) DMAMI (M_1_) with Lim (M_2_) using the penultimate unit model, and (e) DPhAMI (M_1_) with IBVE (M_2_) (triangle) and MorMI (M_1_) with IBVE (M_2_) (square) using the penultimate unit model. *r*_1_ and *r*_2_ are monomer reactivity ratios for the terminal model, and *r*_11_, *r*_21_, *r*_12_, and *r*_22_ are for the penultimate unit model.

**Table 2:** Monomer Reactivity Ratios for the Copolymerization of the AMIs with Vinyl Monomers Controlled by Terminal and Penultimate Unit Models

M_1_	M_2_	*r*_1_	*r*_2_	*r*_1_ × *r*_2_	type of copolymer structure
Terminal Model					
DMAMI	St	0.034	0.050	0.0017	highly alternating
DMAMI	MA	0.69	1.11	0.77	random

M_1_	M_2_	*r*_11_	*r*_21_	*r*_21_/*r*_11_	type of copolymer structure
Penultimate Unit Model					
DMAMI	Lim	0.0050	5.97	1200	AAB-type (strong)
DMAMI	IBVE	0.12	1.81	15	AAB-type (moderate)
MorMI	IBVE	0.11	1.08	9.8	AAB-type (moderate)
DPhAMI	IBVE	0.088	0.11	1.3	AAB-type (weak)/alternating

In contrast to these results, the copolymerization with IBVE could not be analyzed by the terminal model because the calculated composition curves (*r*_1_ = 0.34, *r*_2_ = 0, broken curve) did not agree with the experimental points. On the other hand, the analysis using the penultimate unit model showed good agreement between the experimental points and the calculated composition curve (*r*_11_ = 0.12, *r*_21_ = 1.81, solid curve) (Figure 2c). Here, we set *r*_22_ and *r*_12_ = 0 because IBVE has no homopolymerization reactivity. The obtained *r*_21_ value is 15 times larger than the *r*_11_ value, which indicates that the presence of IBVE as the penultimate unit much favors the addition of the DMAMI monomer to the DMAMI radicals, but the formation of a sequence consisting of successive three DMAMI units is kinetically unfavorable. It is the first report to confirm the penultimate-unit-controlled copolymerization of vinyl ethers with maleimides. In the literature, Lim was reported to exhibit a significant penultimate unit effect in the copolymerization with *N*-phenylmaleimide (PhMI) and its *p*-substituted derivatives.^36,39^ In this study, an intensive penultimate unit effect was observed in the copolymerization of DMAMI with Lim as expected, and the monomer reactivity ratios were determined to be *r*_11_ = 0.0050 and *r*_21_ = 5.97 (Figure 2d).

A similar tendency of the characteristic sequence formation controlled by the penultimate unit effect, i.e., the preferential formation of the AAB sequence, was observed in the copolymerization of IBVE with MorMI (Figure 2e); *r*_11_ = 0.11 and *r*_21_ = 1.08. In contrast, in the copolymerization of DPhAMI containing two bulky phenyl groups in the *N*-amino group, a weak penultimate unit effect was observed (*r*_11_ = 0.088, *r*_21_ = 0.11). The ratio of *r*_21_ to *r*_11_ was close to unity, which was much smaller than that for the copolymerization using DMAMI or MorMI with less steric hindrance. Thus, the penultimate unit effect was relatively small for DPhAMI, and the actually observed reaction fashion was close to that of alternating copolymerization. This is because the alternating AB sequence becomes dominant over the AAB sequence when homopropagation is suppressed by the large steric hindrance of the *N*-substituent, as was reported in the previous paper.^36^ These results clearly show that the chemical structure of the *N*-substituent of the AMIs affects copolymerization reactivity.

The AMIs contain a strong electron-donating amino group as the *N*-substituent. For example, the reported substituent parameters for a dimethylamino group are as follows:^59^ σ_p_ = −0.83, σ_m_ = −0.16, σ* = −0.26 to −0.32, where σ_p_ and σ_m_ are the substituent constants of the Hammett equation and σ* is the substituent constant of the Taft equation. The numerical values of these parameters suggest that the electron density of the double bond in the AMIs is higher than that in *N*-alkylmaleimides and *N*-phenylmaleimides. To discuss this point in more detail, we compared the results of DFT calculations (B3LYP/6-311G* level) together with NMR and UV data for DMAMI, MMI, and PhMI.^60^ The electron density on the double bond carbon, HOMO–LUMO gap, NMR chemical shifts, maximum wavelength of UV absorption (λ_max_), and extinction coefficient (ε) of these maleimides were determined (Table 3). The Mulliken charge of DMAMI indicates that it is the most negatively charged of the three maleimides. It was experimentally confirmed that both the peaks of the carbon and hydrogen atoms of the double bond in the NMR measurements were shifted to a high magnetic field, indicating a high electron density on the double bond. Similar high magnetic field shifts were observed for DPhAMI and MorMI (see NMR data in Supporting Information).

**Table 3:** Molecular Characteristics of Maleimide Monomers with Different *N*-Substituents

		Mulliken charge on C=C	chemical shift^b^ (ppm)		energy level^a^ (eV)		band gap^a^ (eV)	UV (in chloroform)	
maleimide	*N*-sub-stituent	Mulliken charge on C5C	^1^H NMR (CH=CH)	^13^C NMR (C=C)	HOMO	LUMO		λ_max_ (nm)	ε (L/mol cm)
DMAMI	N(CH_3_)_2_	–0.228	6.60	132.7	–6.50	–2.61	3.89	284	612
MMI	CH_3_	–0.226	6.72	134.3	–7.38	–2.62	4.76	283	940
PhMI^c^	C_6_H_5_	–0.219	6.81	134.1	–6.30	–2.82	3.48	280	940

a Based on DFT calculations at the level of B3LYP/6-311G*.

b Measured in CDCl_3_.

c Ref 60.

In general, as the electron-donating property increases, the electron density in the molecule, both HOMO and LUMO, is destabilized and their energy levels increase. However, the HOMO–LUMO gap is often narrowed because the destabilization effect is more pronounced on the HOMO level. On the other hand, the conjugation effect in the molecule acts to energetically stabilize the whole molecule. HOMO level increases and becomes destabilized, while LUMO becomes stabilized. Therefore, it is well known that the HOMO–LUMO gap becomes smaller and the absorption spectrum shifts to longer wavelengths. Comparing the results shown in Table 3, we can see that the HOMO level of DMAMI and PhMI is larger than that of MMI and becomes destabilized. The LUMO level of DMAMI is similar to that of MMI, but the LUMO level of PhMI is decreased (stabilized). These results suggest that the *N*-amino substituent plays little role in the conjugation of the maleimide ring, but the electron-donating effect destabilizes the LUMO and slightly decreases the HOMO–LUMO gap. PhMI showed the smallest band gap due to both conjugation and electron-donating effects. Although the λ_max_ values of these three maleimides are almost the same as those measured by UV measurement, the monomers of DMAMI and PhMI are orange and yellow, respectively, while MMI is colorless. This is because the observed color strongly reflects the difference of the absorption edge at the longer wavelength, i.e., the difference of the band gap.

The electronic properties of these maleimides, estimated by DFT calculations, are expected to induce different characteristics depending on the structure of the *N*-substituent during copolymerization. The characteristics of AMI can be discussed by comparing the copolymerization reactivity ratios for AMI in this study (Table 2) with those of *N*-alkylmaleimides and PhMI and its derivatives reported in the literature (see Table S1). It has already been revealed that the position of the alkyl substituent on the *N*-phenyl group has a significant effect on the copolymerization behavior of PhMI.^36^ It was also pointed out that the solvent effect and polymerization temperature had an important effect on the copolymerization reactivity in the previous reports.^40,42^

From the comparison of monomer reactivity ratios shown in Table 2 and Table S1, the following points can be concluded. There is no drastic change in the copolymerization reactivity of AMI and other monomers, resulting in a similar copolymerization tendency. For example, copolymerization with St produces highly alternating copolymers according to a propagation mechanism controlled by the terminal group in all cases. Copolymerization with isobutene is similarly accounted for by the terminal model, but it is sensitive to the structure of the *N*-substituents and solvents. These systems show higher alternating copolymerization reactivity in halogenated solvents such as chloroform, but lower alternating reactivity in electron-donating solvents such as dioxane or benzene.^37^ This is because a halogenated solvent acts as a Lewis acid, i.e., electron pair acceptor for the carbonyl groups in the maleimide units of monomers and propagating radicals. It has been reported in the copolymerization of PhMI with Lim and β-pinene that the solvent effect has a significant influence on the penultimate unit group effect,^39,40^ and the interaction between the carbonyl groups of monomers and solvents was confirmed experimentally. The copolymerization behavior of AMI may change sensitively depending on the copolymerization conditions. Not only the reaction control of maleimide copolymerization but also the physical property control of sequence-controlled copolymers is expected.

Actually, it has been pointed out in previous studies that the electron-donating property of the *N*-substituent of maleimides favors the expression of the penultimate-unit effect.^36^ This is very consistent with the observation in this study that the AMI copolymerization with IBVE or Lim showed preferred AAB-type sequence formation controlled by the penultimate unit effect. St is one of the typical electron-donating monomers, but in this case, the formation of the AAB-controlled copolymerization propagation was suppressed because of the stronger alternating propagation tendency. In our previous studies,^36^ the copolymerization of *N*-(2,6-dialkyl-substituted phenyl)­maleimides with diisobutene resulted in the formation of the alternating copolymers instead of the AAB-type copolymers. This suggests that the penultimate unit effect is dominated by not only the electron-donating property but also the steric effect.

Several attempts have been made to answer the question of what factors are important for the appearance of the penultimate unit effect during radical copolymerization. However, since the number of copolymerization systems in which the penultimate unit effect was observed was really limited, a clear answer has never been obtained. The introduction of compact and strong electron-donating substituents was expected to be promising for the induction of the penultimate unit effect during copolymerization, but this hypothesis could not be proved experimentally because the electron-donating properties of *N*-alkyl and *N*-phenyl groups were limited. In this study, the introduction of strong electron-donating amino groups directly onto the maleimide ring without a spacer enabled sequence control by the penultimate unit effect during copolymerization with IBVE and Lim as the nonconjugating and electron-donating monomers. Furthermore, the effect of the *N*-substituent on the amino group has been clarified. It has also been found that the AMIs containing *N’,N’*-dimethylamino, *N’,N’*-diphenylamino, and morpholino groups as the *N*-substituents show copolymerization properties depending on the steric hindrance of the substituents.

### Monomer Synthesis Using Mono-Substituted Hydrazine

In this study, the AMIs were synthesized using three kinds of *N,N*-disubstituted hydrazines as one of starting materials according to the procedure described in Scheme 3.^1^ From the comparison of the copolymerization reactivities of the obtained AMIs, it has been clarified that the chemical structure of the *N*-amino groups (i.e., -NR^1^R^2^ group in Scheme 3) affects the copolymerization rate, the monomer reactivity ratio, and the magnitude of the evolving penultimate unit effect. It was speculated that the copolymerization behavior would be more significantly affected by the structure of the *N*-substituent on the maleimide ring, if any maleimide with a mono-substituted amino group, i.e., -NHR^1^ substituent, was synthesized using an *N*-mono-substituted hydrazine. In this study, *N*-(*N′*-phenylamino)­maleimide (PhAMI) was tried to prepare by the same procedure as that of the AMI synthesis using *N′,N′*-disubstituted hydrazines.^49–52^ The reaction of maleic anhydride with *N*-phenylhydrazine was confirmed to give the corresponding *N*-(*N′*-phenylamino)­maleamic acid by NMR spectroscopy (Figure S8). After the second-step cyclization was carried out, the ^1^H and ^13^C NMR spectra of the isolated product were checked. The product indicated that cyclization had occurred, but two kinds of peaks based on the C=C carbons were observed (Figures S9 and S11), inconsistent with what would be expected from the symmetric chemical structure of PhAMI. It was pointed out that older literature contained errors regarding the structural determination and identification of PhAMI and the related compounds.^49,61–66^ As a result, reliable synthetic routes for PhAMI and the others have not been established yet. Therefore, we carefully identified the exact chemical structure of the product obtained in this study.

**Scheme 3:**
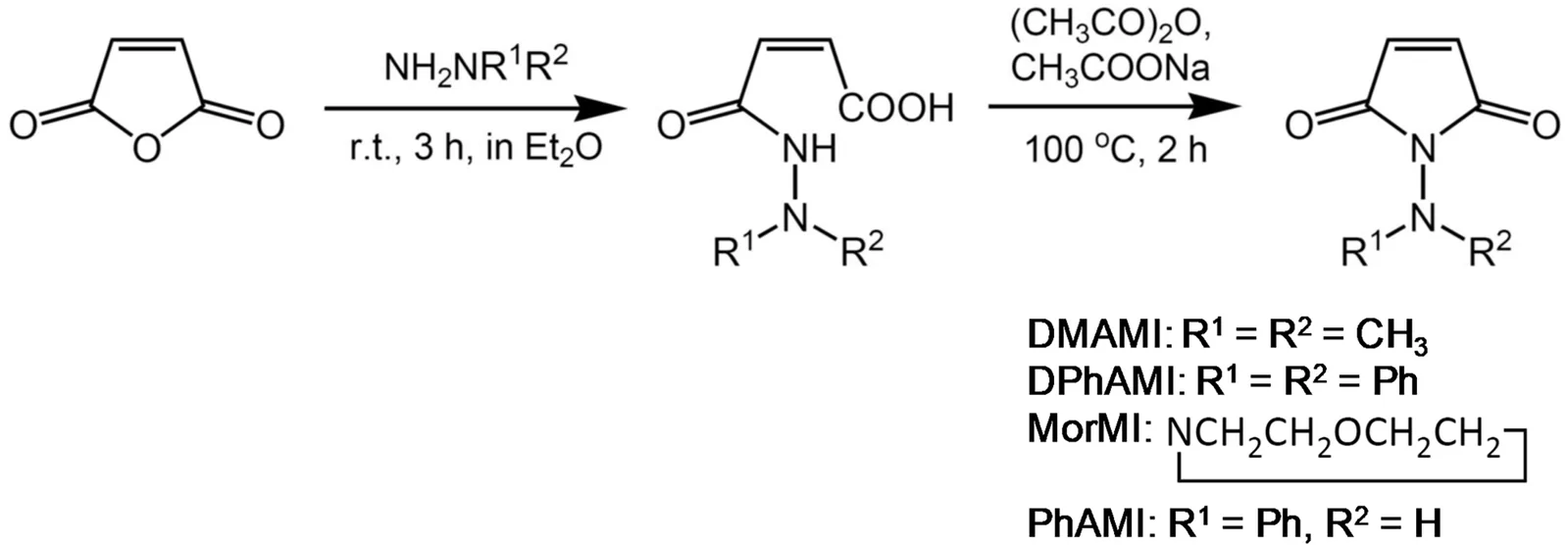
Typical Reaction Pathway for the Synthesis of AMIs Using Maleic Anhydride as the Starting Material^a^

a Sodium acetate and acetic anhydride are often used for the intramolecular condensation to produce 5-membered cyclic products.

Judging from the features of the carbonyl carbon and other absorption peaks, it was concluded that a 6-membered cyclic compound B with an asymmetric ring structure was formed, as shown in Scheme 4. Here, the broad peak around 8.4 ppm in the ^1^H NMR spectrum disappeared by an exchange reaction with CD_3_CD (Figure S10). It is assumed that the cyclization of *N*-(*N′*-phenylamino)­maleamic acid did not form the desired 5-membered ring but gave a 6-membered ring compound. The product A shown in Scheme 4 is a compound called maleimidohydrazine, of which synthesis and properties have been reported in the literature.^49^ Several reports have pointed out that maleimidohydrazines are thermally unstable and their isomerization proceeds under mild conditions to give the corresponding keto-enol tautomer, product B.^49,63,64,67^ To investigate whether the isomerization of product A to product B actually proceeds, DFT calculations (B3LYP/cc-PVTZ level) were performed on the compounds formed by the reaction route shown in Scheme 4. As a result, relative heat values for the formation of product A and product B, which were determined based on the heat of formation of PhAMI (Δ*E* = 0), were ΔΔ*E*_A_ = −10.69 kJ/mol and ΔΔ*E*_B_ = −21.03 kJ/mol, respectively. This result strongly supports that the structure of PhAMI is thermodynamically unfavorable and that the most energetically stable compound B was actually isolated in this study.

**Scheme 4:**
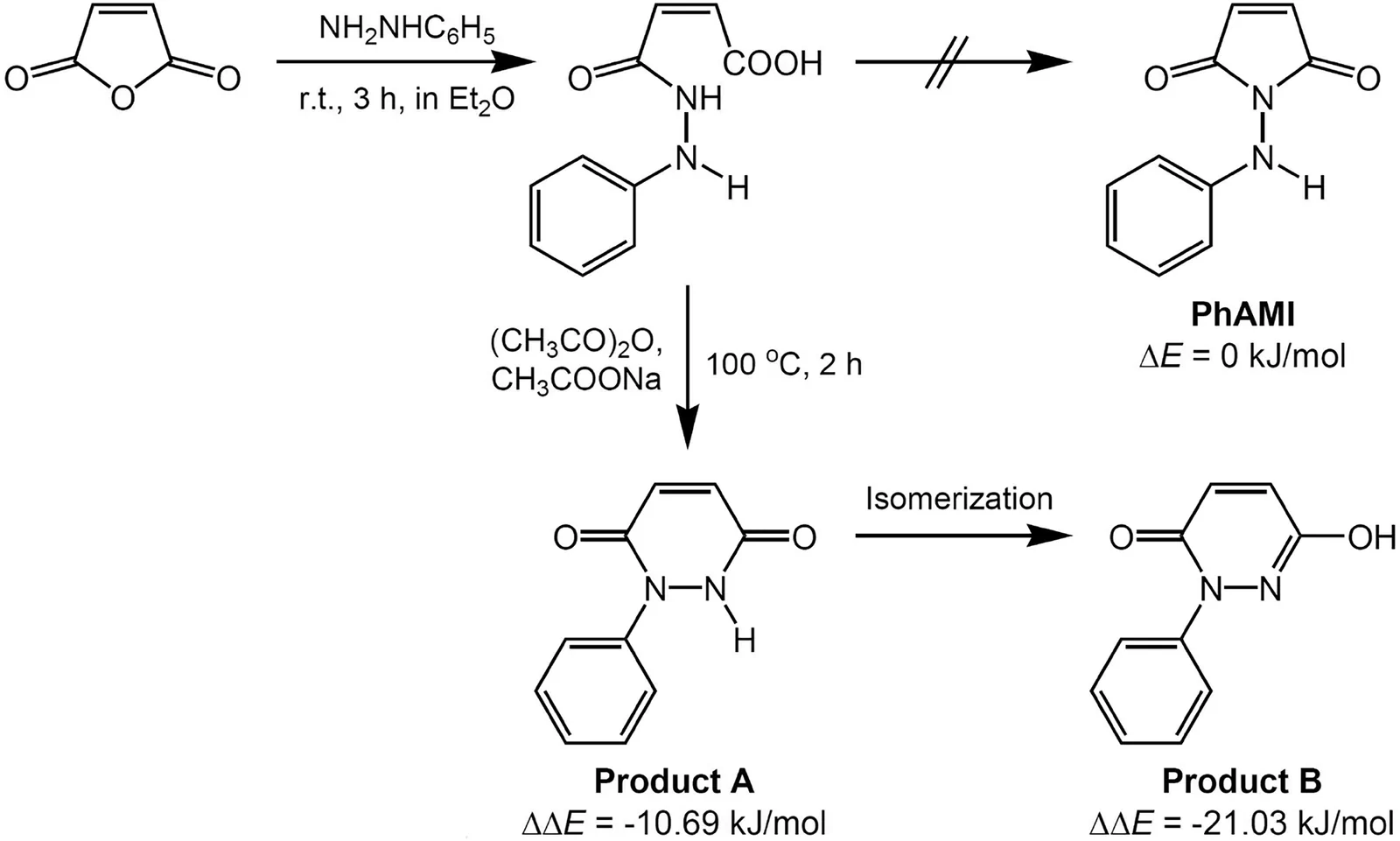
Attempt to Synthesize *N*-(*N‘*-Phenylamino)­maleimide (PhAMI) and the Actually Observed Reaction Pathway to Yield a 6-Membered Cyclic Product A as the Intermediate Product, Which Further Provided Another Structural Isomer B (Keto-Enol Tautomer)

When copolymerization of product B with St was attempted under the reaction conditions of 1/1 molar ratio in CDCl_3_ at 60°C for 5 h, no polymer was produced. The NMR measurement of the solution after heating showed no change in the integral values of product B and St with respect to the internal standard, indicating that neither monomer was consumed (Figure S12). Even when a small amount of product B (for example, 5 wt % of St) was added to a solution polymerization system of St, no polymer was formed. This indicates that the presence of product B effectively inhibits radical polymerization. In order to verify the possibility of hydrogen abstraction from product B for inhibiting the polymerization, the changes in the heat of formation were estimated for radical addition to product B and hydrogen abstraction using DFT calculations (Scheme 5). Here, calculations were performed for two sets of reactions involving methyl and hydroxy radicals, respectively. The results are shown in Table 4. The energy difference between the addition of a methyl radical to the double bond of product B and the hydrogen abstraction from product B was Δ*E*_abst_ – Δ*E*_add_ = −11.04 kJ/mol. This indicates that hydrogen abstraction is thermodynamically more favorable than radical addition. For the hydroxy radical reactions, the energy difference was much larger (−40.37 kJ/mol), which is consistent with the generally observed tendency that an oxygen-centered radical tends to abstract hydrogen. From these DFT calculations, it was concluded that the radical formed from product B was strongly resonance-stabilized and had a significant effect in inhibiting radical polymerization. It should be noted that the superior inhibition ability toward a St radical completely differs from the difficulty of the complete inhibition of the St polymerization using phenol derivatives such as catechol and 2,6-di-*tert*-butyl-4-methylphenol.^68,69^ Thus, it has been found that product B is not suitable to be used as a monomer, but rather to be applied as a strong polymerization inhibitor. The results of this study suggest that the stabilizing effect against the thermal and oxidative degradation of commodity polymers by adding any aminomaleimide derivative, as reported in the literature,^70–72^ may be due to the action of stable radicals.

**Scheme 5:**
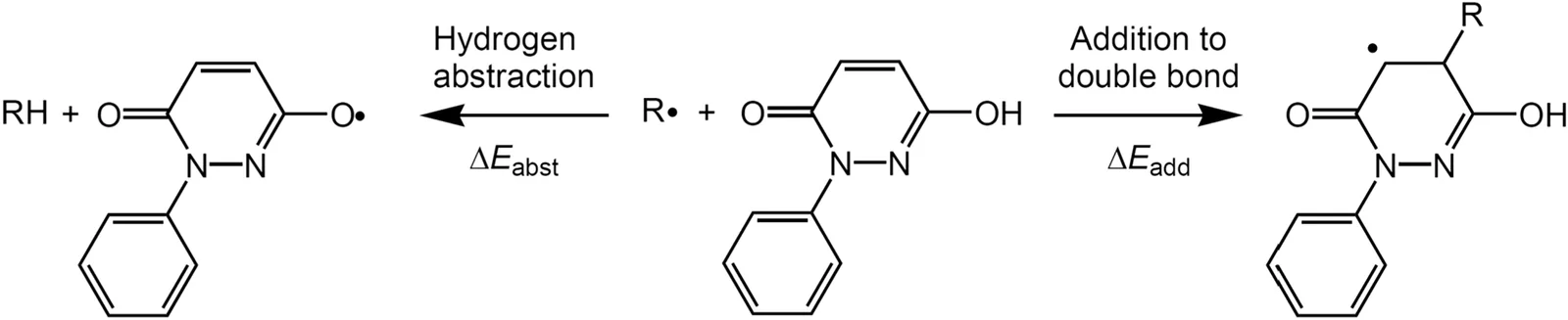
Estimation of Thermodynamic Stability of the Radicals Produced from the 6-Membered Compound B by Hydrogen Abstraction and Addition to the Double Bond of Radicals

**Table 4:** Enthalpy Changes in the Reactions of the 6-Membered Compound B Radicals with Methyl and Hydroxy Radicals by Hydrogen Abstraction and Addition to the Double Bond^a^

radical	Δ*E*_abst_ (kJ/mol)	Δ*E*_add_ (kJ/mol)	Δ*E*_abst_ – Δ*E*_add_ (kJ/mol)
^•^CH_3_	–89.11	–78.07	–11.04
^•^OH	–131.53	–91.16	–40.37

a Determined by DFT calculations at the level of B3LYP/cc-pvTZ.

### Thermal Properties of Copolymers

In general, when organic polymers are heated in the presence of oxygen, exothermic decomposition reactions, such as oxidation and combustion, take precedence. While under an inert atmosphere, decomposition proceeds by endothermic reaction processes, such as carbon-to-carbon bond scission. In contrast, it has been reported that polymers containing hydrazine bonds are easily oxidized and decompose exothermally in an inert atmosphere.^73–76^ Since the copolymer synthesized in this study was expected to undergo exothermic decomposition, the thermal decomposition behavior of the copolymer containing the AMI units was investigated by TGA/DTA analysis.

The 10% weight-loss temperature (*T*_d10_), maximum decomposition temperature (*T*_max_), weight residue at 500 °C (*CY*_500_), and enthalpy of decomposition (Δ*H*) of the copolymers, determined at a heating rate of 10°C/min under nitrogen or air gas flow, are summarized in Table 5. As shown in typical TGA and DTA curves for the DMAMI/MA copolymers in Figure 3(a),(b), the copolymers were stable up to approximately 300°C and showed a rapid weight loss over 300°C. As shown in the DTA curves, the decomposition accompanied by the weight loss was exothermic when a large amount of the AMI units were introduced into the copolymers. As shown in Table 5, both *T*_d10_ and *T*_max_ values decreased and *CY*_500_ increased with increasing DMAMI content when it was less than 50 mol %. For the copolymers with St or IBVE, it is difficult to obtain copolymers with largely different compositions because alternating or AAB repeating is preferred. The copolymers containing 48–67 mol % of the AMI units showed similar decomposition behavior, and *T*_d10_ of the copolymers was above 300°C roughly regardless of the type of M_1_ or M_2_.

**Figure 3:**
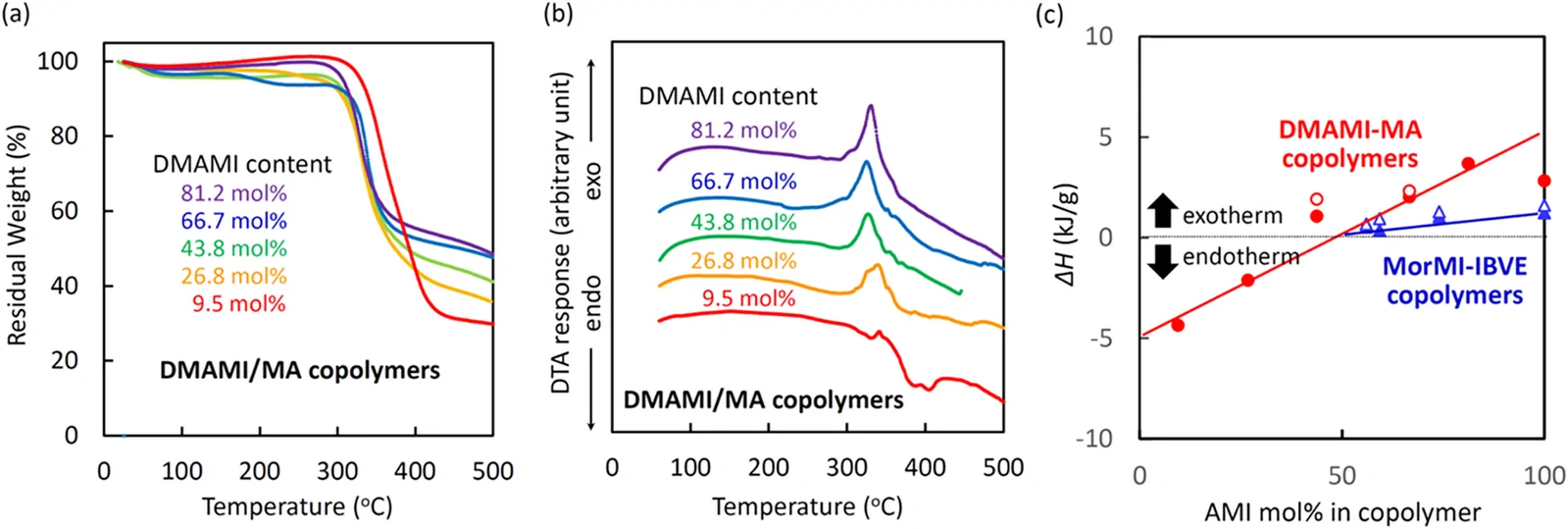
(a) TGA and (b) DTA curves for the DMAMI/MA copolymers in nitrogen stream at the heating rate of 10°C/min. (c) Relationship between the AMI content and the Δ*H* value of the copolymers. Closed symbols: in N_2_, open symbols: in air, red circles: DMAMI/MA copolymers, blue triangles: MorMI/IBVE copolymers.

**Table 5:** Thermal Decomposition Parameters for the Copolymers of AMI (M_1_) with Vinyl Monomers (M_2_)

M_1_	M_2_	M_1_ mol % in copolymer	TGA^a^ (10 °C/min, N_2_)				DSC (10 °C/min)
			*T*_d10_ (°C)	*T*_max_ (°C)	*CY*_500_ (%)	Δ*H* (kJ/g)	*T*_g_ (°C)
DMAMI	St	47.7	342	393	14.8		185
DMAMI	St	56.7	348	394	11.5		204
DMAMI	St	67.2	345	392	17.7		217
DMAMI	MA	9.5	350	416	17.6	–4.36	38
DMAMI	MA	26.8	340	358	29.8	–2.12	87
DMAMI	MA	43.8	312	336	35.9	1.06 [1.91]	121
DMAMI	MA	50.0	313	328	26.5		130
DMAMI	MA	66.7	313	326	41.0	2.03 [2.32]	187
DMAMI	MA	81.2	317	329	48.4	3.68	
DMAMI		100	301	346	45.1	2.83	>300
DMAMI	IBVE	58.1	302	317	39.1		135
DPhAMI	IBVE	56.5	326 [324]	337 [337]	23.0 [34.5]	0.32 [0.83]	175
MorMI	IBVE	56.0	307 [304]	326 [333]	27.7 [29.7]	0.53 [0.67]	
MorMI	IBVE	59.3	308 [310]	326 [331]	29.2 [27.8]	0.36 [0.95]	177
MorMI	IBVE	74.0	292 [304]	330 [330]	29.1 [39.0]	1.04 [1.29]	236
MorMI		100	248 [234]	347 [348]	32.9 [28.6]	1.24 [1.61]	

a Values in parentheses indicate the data determined in air.

Although gaseous components as the decomposition products were not analyzed in this study, the following decomposition reactions can be inferred. First, bond scission occurs around the hydrazine structure in the side chain of the copolymer, followed by complicated reactions with the generation of N_2_ gas and other volatile decomposition products. Generally, vinyl polymers decompose endothermically in inert gases, but the decomposition enthalpy value of the AMI copolymers increased with increasing AMI content. The energy balance of the entire thermal decomposition reaction switched from endothermic to exothermic at around 50 mol % of the AMI content (Figure 3(c)). In addition, the decomposition temperature and enthalpy change were only slightly influenced by the atmospheric conditions, i.e., in N_2_ or air. Similar exothermic decomposition was observed for the copolymers of DPhAMI and MorMI. Exothermic decomposition of nitrogen-rich materials, such as polymers containing N–N bonds, has been reported in the literature.^73,74^ Furthermore, although it is a preliminary experimental result, it has been confirmed that the copolymers containing AMI units undergo oxidative decomposition as well as other polymers containing N–N structural units^75,76^ (Figure S24 and Video S1). Therefore, it is expected that the copolymers will be applied as new degradable and reactive polymers in the future.

Figure 4 shows the effect of the DMAMI content on *T*_g_ of the copolymers with MA, St, and IBVE. The *T*_g_ values observed for each copolymer are summarized in Table 5. In Figure 4, curves calculated by Fox’s equation (eq 1) are also depicted.^77^

1 $$1/T_{g}=\left(w_{A}/T_{g,A}\right)+\left(\left(1-w_{A}\right)/T_{g,B}\right)$$

where *T*_g_, *T*_g,A_, and *T*_g,B_ are *T*_g_ of the copolymer, homopolymer A, and homopolymer B, respectively, and *w*_A_ is the weight fraction of A component in the copolymer. The *T*_g_ values for the homopolymers of St, MA, and IBVE are 100°C, 8°C, and −22 °C, respectively.^78^ Since the *T*_g_ of the homopolymer of DMAMI could not be accurately determined due to the onset of thermal decomposition, the curve is drawn assuming that the *T*_g_ of the homopolymer of DMAMI is 300 °C. For the copolymers with MA and St, *T*_g_ increases with an increasing DMAMI content. It indicates that the molecular motion of the copolymers is suppressed with the increasing DMAMI content. As shown in the figure, the copolymers of DMAMI and MA have a random arrangement structure, and the *T*_g_ can be precisely adjusted in a wide temperature range depending on the feed composition. For the copolymers with St and IBVE, the measured *T*_g_ values similarly agreed with those predicted from the equations.

**Figure 4:**
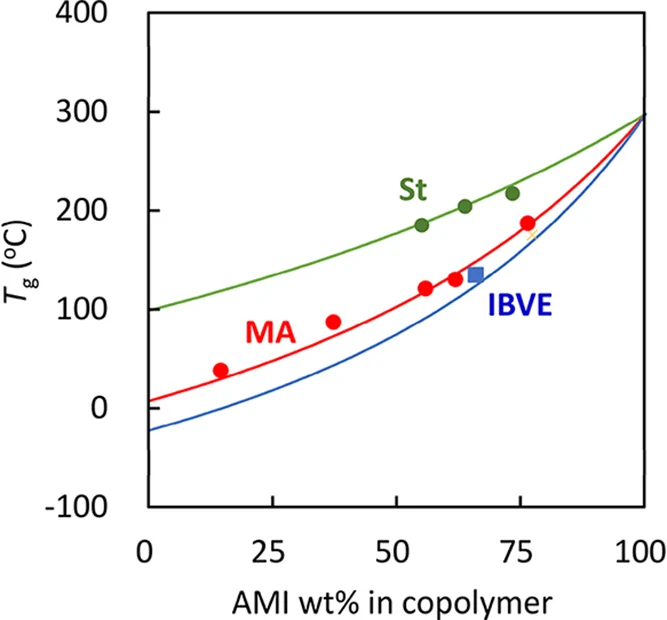
Composition dependence of *T*_g_ values for the copolymers of DMAMI with MA, St, and IBVE. The curves are depicted according to the Fox equation. The *T*_g_ values for the homopolymers of St, MA, and IBVE are assumed to be 100 °C, 8 °C, and −22 °C, respectively.

## Conclusion

In this study, the AMIs containing an *N’,N’*-disubstituted amino group as the *N*-substituent were synthesized from maleic anhydride and *N,N*-disubstituted hydrazine compounds, and their radical copolymerization with various vinyl monomers was carried out. The composition and arrangement of the repeating unit structure as well as the thermal properties and decomposition behavior of the resulting copolymers were investigated. The radical copolymerization of DMAMI with MA gave random copolymers, and the copolymerization with St gave highly alternating copolymers. During the copolymerization, the propagation was controlled by a mechanism according to the terminal model. On the other hand, the copolymerization with IBVE or Lim was controlled by the penultimate unit model, and consequently, AAB-type repeating units were preferred. It was also clarified that the introduction of compact and electron-donating *N*-substituents is promising for the induction of the penultimate unit effect. The 10% weight-loss temperatures of the copolymers were over 300°C, and the copolymers containing a large amount of the AMI units decomposed exothermically irrespective of the decomposition atmosphere. The *T*_g_ of the copolymers varied over a wide temperature range depending on the structure and composition of the copolymer sequences, being consistent with the values predicted from Fox’s equation. In addition, we tried to synthesize *N*-(phenylamino)­maleimide, but the actually isolated product was another 6-membered ring compound. It was found that the isolated compound showed no polymerization reactivity, but rather inhibited polymerization by a small amount upon addition. The decomposition temperature of the AMI copolymers is sufficiently high and stable at room temperature, and it can be handled in the same conventional way as that for commodity polymers. In recent years, the design of degradable polymer materials, including biodegradable polymers, has attracted much attention. The copolymer synthesized in this study is a new type of polymer material with a high decomposition temperature, a tunable *T*_g_ in a wide temperature range, and an exothermic degradation property at a higher temperature. As a result of the preliminary study, it was also confirmed that oxidative degradation of the AMI copolymers is possible. The unique thermal and degradation properties of the AMI copolymers open new opportunities for the design of advanced functional polymer materials with controllable stability and degradability.

## Supplementary Material





## Notes

Additional experimental details, method for copolymerization analysis, NMR, UV–vis, and IR spectra, and oxidative decomposition (PDF)
Copolymers containing AMI units undergo oxidative decomposition as well as other polymers containing N–N structural units (MP4)
