# Supplementary material for: Precise Sequence Control and Thermal Characterization of N‑Substituted Maleimide Copolymers Containing Hydrazine Structure
Source: Polym Sci Technol. 2026 Jul 6;2(10):824–35. doi: 10.1021/polymscitech.6c00081 (PMC13718368; doi:10.1021/polymscitech.6c00081)
Supplement: Supplementary file 1 [file ps6c00081_si_001.pdf]

Supporting Information for *Polymer Science & Technology*

Precise Sequence Control and Thermal Characterization of *N*-Substituted Maleimide Copolymers Containing Hydrazine Structure

Akikazu Matsumoto,[1*] Natsuki Okada,[2] Yuki Nakai,[2] Tomohiro Suzuki,[2] and Yasuhito Suzuki[1*]

[1] *Department of Applied Chemistry, Graduate School of Engineering, Osaka Metropolitan University, 1-1, Gakuen-cho, Naka-ku, Sakai, Osaka 599-8531, Japan*
[2] *Department of Applied Chemistry, Faculty of Engineering, Osaka Prefecture University, 1-1, Gakuen-cho, Naka-ku, Sakai, Osaka 599-8531, Japan*

*** Corresponding authors.**
E-mail address: akimatsumoto@omu.ac.jp (Akikazu Matsumoto) and suzuki_y@omu.ac.jp (Yasuhito Suzuki)

Contents

1. Experimental section

1.1. Reagents

1.2. Apparatus

1.3. Monomer synthesis

1.4. Polymerization method

1.5. Evaluation of polymerization reactivity of product B

2. Analysis of copolymerization

2.1. Composition analysis of copolymers

2.2. Determination of monomer reactivity ratio

3. NMR spectra

4. UV-vis spectra

5. IR spectra

6. Oxidative decomposition of the AMI copolymer

7. Monomer reactivity ratios reported in the literature

8. References

1. Experimental section

1.1. Reagents

Maleic anhydride (Nacalai Tesque Co., Ltd.), sodium acetate (Nacalai Tesque Co., Ltd.), acetic anhydride (Tokyo Kasei Kogyo Co., Ltd. (TCI)), 1,1-dimethylhydrazine (TCI), phenylhydrazine (TCI), 1,1-diphenylhydrazine hydrochloride (TCI), *N*,*N'*-dicyclohexylcarbodiimide (Nacalai Tesque Co., Ltd.), 4-(*N,N*-dimethylamino)pyridine (Nacalai Tesque Co., Ltd.), pentaphosphorus dioxide (Fujifilm Wako Pure Chemical Industries, Ltd.), concentrated sulfuric acid (Fujifilm Wako Pure Chemical Industries, Ltd.), 1,4-dioxane (Fujifilm Wako Pure Chemical Industries, Ltd.), dimethylformamide (Nacalai Tesque Co., Ltd.), *n*-hexane (Nacalai Tesque Co., Ltd.), methyl acrylate (MA, TCI), and isobutyl vinyl ether (IBVE, Sigma-Aldrich Co., Ltd.) were used without further purification from commercial sources. Styrene (St, Nacalai Tesque Co., Ltd.) was distilled under reduced pressure. 2,2'-Azobis(isobutyro-nitrile) (AIBN) was purchased from Fujifilm Wako Pure Chemical Industries, Ltd., and recrystallized with *n*-hexane. Chloroform-*d* and methanol-d_4 were purchased from Fujifilm Wako Pure Chemical Industries, Ltd., and used as solvents for NMR measurements.

1.2. Apparatus

^{1}H NMR and ^{13}C NMR spectra were measured using an Ascend 400 Bruker Biospin. As One ATM-01 was used for melting point measurements. Size exclusion chromatography (SEC) measurements were performed using JASCO LC-NetII/ADC, CO-2065Plus, UV-2070Plus, RI-2031Plus, PU-2080Plus, and DG-2080-53. Thermogravimetric and differential thermal analyses (TGA/DTA) and differential scanning calorimetry (DSC) measurements were performed using DTG-60 and DSC-60 manufactured by Shimadzu Corporation, respectively (Nitrogen or air flow rate 100 mL/min, heating rate 10°C/min). DFT calculations were performed using Spartan'18. The calculation level was B3LYP/6-311G* or B3LYP/cc-pvTZ.

1.3. Monomer synthesis

1.3.1. Synthesis of *N*-(*N'*,*N'*-dimethylamino)maleimide (DMAMI)

DMAMI was synthesized according to the following procedure. First, 3.88 g (0.04 mol) of maleic anhydride was dissolved in 100 mL of diethyl ether in a 100 mL round-bottom flask, and then 3.0 mL (0.04 mol) of *N,N*-dimethylhydrazine was added. The mixture was stirred at room temperature for 2 h to obtain *N*-(*N',N'*-dimethylamino)maleamic acid as a yellow viscous substance. The solvent was

removed by evaporator, and the mixture was used as it was in the next reaction without purification (**Figure S1**). To a 100 mL round-bottom flask containing the synthesized *N*-(*N′,N′*-dimethylamino)-maleamic acid was added 24 mL of acetic anhydride and 1.33 g of sodium acetate, and the mixture was heated at 100°C for 2 h to reflux. After allowing to cool to room temperature, distilled water was added, and the mixture was stirred in an ice bath for 2 h. The mixture was extracted with diethyl ether and purified by silica gel column chromatography (chloroform/*tert*-butyl methyl ether = 9/1). Yield 0.80 g (16%), melting point 73°C, yellow crystals. ^{1}H NMR (400 MHz, $CDCl_3$) δ6.60 (s, 2H), 2.93 (s, 6H) (**Figure S2**); ^{13}C NMR (100 MHz, $CDCl_3$) δ169.1, 132.7, 45.0 (**Figure S3**); IR (neat on KBr plate) 1735, 1724, 1590, 1495, 1230, 1150, 823, 748, 691, 632 cm^{-1} (**Figure S23**). NMR data reported in the literature:[1)] ^{1}H NMR (400 MHz, $CDCl_3$) δ6.78 (s, 2H), 2.71 (s, 6H); ^{13}C NMR (100 MHz, $CDCl_3$) δ174.1, 134.9, 47.3.

1.3.2. Synthesis of *N*-(*N′*,*N′*-diphenylamino)maleimide (DPhAMI)

DPhAMI was synthesized by a procedure similar to that for DMAMI. 1.32 g (0.015 mol) of maleic anhydride was placed in a 200 mL round-bottom flask, and dissolved in 20 mL of dimethylformamide. *N,N*-Diphenylhydrazine hydrochloride (1.46 g, 0.015 mol) dissolved in 100 mL of dimethylformamide was added to the mixture, and the mixture was stirred at room temperature for 2.5 h. The formation of *N*-(*N′*,*N′*-diphenylamino)maleamic acid was confirmed by NMR spectroscopy. Sodium acetate (0.657 g) and acetic anhydride (10 mL) were added to this solution, and the mixture was heated and stirred at 80°C for 2 h. Allow to cool to room temperature, add 100 mL of distilled water, and stir in an ice bath for 2 h. Extract with toluene, remove the solvent under reduced pressure, and purify by silica gel column chromatography (chloroform/acetone = 9/1).

Yield 1.47 g (37%), melting point 137°C, yellow crystals. ^{1}H NMR (400 MHz, $CDCl_3$) δ7.32-7.26 (m, 5H), 7.10-7.07 (m, 5H), 6.79 (s, 2H) (**Figure S4**); ^{13}C NMR (100 MHz, $CDCl_3$) δ168.3, 144.5, 132.7, 129.5, 124.1, 120.2 (**Figure S5**); IR (neat on KBr plate) 1726, 1170, 838, 728 cm^{-1} (**Figure S23**). NMR data reported in the literature:[1)] ^{1}H NMR (400 MHz, $CDCl_3$) δ7.45-7.41 (m, 4H), 7.36-7.26 (m, 2H), 7.23-7.15 (m, 3H), 7.13-7.10 (m, 1H), 6.79 (s, 2H); ^{13}C NMR (100 MHz, $CDCl_3$) δ171.2, 142.6, 138.1, 131.2, 124.3, 122.0.

1.3.3. Synthesis of *N*-morpholinomaleimide (MorMI)

MorMI was synthesized by a procedure similar to that for DMAMI. In a 100 mL round-bottom

flask, 0.98 g (0.01 mol) of maleic anhydride was dissolved in 100 mL of chloroform. To this solution, 1.0 g (0.01 mol) of 4-aminomorpholine was added, and the mixture was stirred at room temperature for 10 h to obtain the desired product. A part of the solvent was removed under reduced pressure with an evaporator to concentrate the solution, which was used for the next step. To a 100 mL round-bottom flask containing 2.7 g of compound including *N*-morpholinomaleamic acid, 20 mL of acetic anhydride and 0.82 g of sodium acetate were added, and the mixture was heated to reflux at 90°C for 3 h. After allowing to cool to room temperature, 30 mL of distilled water was added, and the mixture was stirred in an ice bath for 2 h. Then, the mixture was extracted with chloroform, and the chloroform was lightly removed with an evaporator. Purification was carried out by silica gel column chromatography (*n*-hexane/ethyl acetate = 4/6). It was dissolved in warmed *n*-hexane and cooled in a freezer overnight for recrystallization. It was dried in vacuum for 3 h to obtain MorMI. Yield 0.32 g (18%), melting point 95°C, yellow crystals. ^{1}H NMR (400 MHz, $CDCl_3$) δ6.62 (2, 2H), 3.85-3.82 (t, J = 6 Hz, 4H), 3.31-3.29 (t, J = 6 Hz, 4H) (**Figure S6**); ^{13}C NMR (100 MHz, $CDCl_3$) δ168.8, 132.6, 67.0, 52.5 (**Figure S7**); IR (neat on KBr plate) 1735, 1450, 1356, 1180, 1148, 1150, 1027, 850, 772, 695 cm^{-1} (**Figure S23**). NMR data reported in the literature: [1)] ^{1}H NMR (400 MHz, $CDCl_3$) δ6.75 (s, 2H), 3.78 (t, J = 3.9 Hz, 4H), 2.95 (t, J = 4.6 Hz, 4H); ^{13}C NMR (100 MHz, $CDCl_3$) δ171.2, 133.6, 65.3, 54.8.

1.3.4. Attempt to synthesize *N*-(*N′*-phenylamino)maleimide (PhAMI)

Maleic anhydride (3.97 g, 0.04 mol) and dimethylformamide (20 mL) were added to a 100 mL round-bottom flask and dissolved by stirring. *N*-Phenylhydrazine (4.0 mL, 0.04 mol) was added, and stirring was continued for 3 h. The solution was poured into ice water and allowed to stand for a while. The yellow powder precipitated at the bottom was collected by suction filtration, and heated at 110°C for 30 min to evaporate the solvent under reduced pressure. The structure of the produced *N*-(*N′*-phenylamino)maleamic acid was checked by ^{1}H NMR spectroscopy (**Figure S8**). Recrystallization purification was not performed, and the crude product was used as it was in the next reaction.

The synthesis of PhAMI was attempted under the following conditions. *N*-(*N′*-Phenylamino)-maleamic acid (4.12 g), sodium acetate (3.25 g), and acetic anhydride (60 mL) were placed in a 200 mL round-bottom flask, and heated to reflux at 80°C for 2 h. After allowing to cool to room temperature, 100 mL of distilled water was added, and the mixture was stirred in an ice bath for 2 h. The obtained solution was poured into the ice bath, and the precipitate was collected by suction filtration, and washed 3 times with distilled water. The obtained crystal was dried under reduced

pressure. The structure of the isolated compound was determined to be of product B shown in **Scheme 4** in the text (**Figures S9-11**). The isolated yield of product B was not determined in this study. ^{1}H NMR (400 MHz, $CDCl_3$) δ8.21(br, 1H), 7.39 (d, J = 5 Hz, 1H), 7.34-7.29 (m, 2H), 7.13 (d, J = 5 Hz, 2H), 7.01-6.96 (t, J = 5 Hz, 1H), 6.29 (d, J = 5 Hz, 1H); ^{1}H NMR (400 MHz, CD_3OD) δ7.57 (d, J = 5 Hz, 1H), 7.29-7.22 (m, 4H), 6.94-6.90 (m, 1H), 6.37 (d, J = 5 Hz, 1H); ^{13}C NMR (100 MHz, $CDCl_3$) δ166.4, 142.5, 141.3, 140.0, 129.5, 122.2, 113.3. NMR data for product B reported in the literature: [2,3)] ^{13}C NMR (50.3 MHz, dimethyl sulfoxide-d_6) δ157.9, 152.9, 141.7, 134.1, 128.5, 127.7, 127.5, 125.4; ^{1}H NMR (dimethyl sulfoxide-d_6) δ7.68-7.25 (m, 5H), 7.28 (d, J = 10 Hz, 1H), 7.0 (d, J = 10 Hz, 1H). The synthetic procedure and NMR data for PhAMI reported in the literature: [4)] mp 145-148°C; ^{1}H NMR (dimethyl sulfoxide-d_6, 300 MHz) δ7.79 (d, J = 3.0 Hz, 1H), 7.28-7.24 (m, 4H), 6.89-6.86 (m, 1H), 6.55 (d, J = 3.0 Hz, 1H); ^{13}C NMR (dimethyl sulfoxide-d_6) δ167.0, 143.7, 140.8, 129.1, 121.0, 119.7, 113.3; Hiran et al. also reported the following NMR data,[5)] but the assignment of the NMR spectrum and the identification of the compound may contain errors (See also the report by Conley et al.:[6)] mp 215°C; ^{1}H NMR ($CDCl_3$, 300 MHz) δ7.5-6.8 (m, 4H, phenyl), 3.74 (s, 1H, N-H), 6.3 and 7.1 (2H, s, O=C-CH=CH-C=O).

1.4. Polymerization method

Predetermined amounts of monomers and AIBN were dissolved in chloroform and transferred to a polymerization glass tube. Freezing, degassing, and thawing cycles were repeated three times, and finally nitrogen was sealed. After polymerization in a bath kept at 60°C for a predetermined time, the polymer was precipitated by adding a large amount of *n*-hexane. The polymer isolated by filtration or decantation was purified by reprecipitation with an appropriate solvent and methanol.

1.5. Evaluation of polymerization reactivity of product B

The $CDCl_3$ solution of product B and St containing AIBN and a small amount of 1,4-dioxane was prepared at the conditions of [product B] = [St] = 0.15 mol/L and [AIBN] = 3 mmol/L. Here, 1,4-dioxane was used as an internal standard. After bubbling with nitrogen for 5 min, the polymerization was tried at 60°C. After 5 h of the reaction, ^{1}H NMR measurement was performed to evaluate the progress of polymerization (**Figure S12**).

2. Analysis of copolymerization

2.1. Composition analysis of copolymers

The composition of copolymers was calculated based on the ratio of the integral values of the characteristic peaks of each copolymer in the ^{1}H NMR spectra (**Figures S13-17**). For the DMAMI/MA and DMAMI/IBVE copolymers, the composition was estimated based on the integral ratio of the hydrogens of the side chain and the main chain. For the DMAMI/St copolymers, the composition was estimated based on the integral ratio of the aromatic hydrogens and the others. For the other copolymers, the composition was determined similarly. In addition, under the same polymerization conditions, the polymerization solution in $CDCl_3$ was subjected to ^{1}H NMR spectroscopy at predetermined intervals. Time-conversion curves of each monomer were prepared using 1,4-dioxane as an internal standard. The composition of copolymers was estimated based on the ratio of the consumption rates of the both monomers, which were determined from the initial slopes of a kinetic plot for first-order reaction.

2.2. Determination of monomer reactivity ratios

The propagation reactions of copolymerization according to the terminal model are shown below.

$$\sim\sim M_1\bullet + M_1 \xrightarrow{k_{11}} \sim\sim M_1\bullet$$

$$\sim\sim M_1\bullet + M_2 \xrightarrow{k_{12}} \sim\sim M_2\bullet$$

$$\sim\sim M_2\bullet + M_1 \xrightarrow{k_{21}} \sim\sim M_1\bullet$$

$$\sim\sim M_2\bullet + M_2 \xrightarrow{k_{22}} \sim\sim M_2\bullet$$

Here, M_1 and M_2 represent two kinds of monomers 1 and 2, and ~~M_1· and ~~M_2· represent propagating radicals, respectively. The comonomer-copolymer composition relationship is represented by the following Mayo−Lewis equation.

$$\frac{\mathrm{d}[\mathrm{M}_1]}{\mathrm{d}[\mathrm{M}_2]} = \frac{[\mathrm{M}_1]\left(r_1[\mathrm{M}_1]+[\mathrm{M}_2]\right)}{[\mathrm{M}_2]\left(r_2[\mathrm{M}_2]+[\mathrm{M}_1]\right)} \quad \text{(S1)}$$

Here, the monomer reactivity ratios, r_1 and r_2, are $r_1 = k_{11}/k_{12}$ and $r_2 = k_{22}/k_{21}$, respectively.

They can be estimated by the Fineman−Ross and Kelen−Tüdős methods, but these methods often give scattered plots and the comonomer−copolymer composition curves based on the calculated values partially failed to fit the experimental data. In contrast, the curve fitting method results in good results over the whole range of compositions for the copolymerization systems of the RMIs,

as was shown in previous papers.[7,8)]

On the other hand, the penultimate unit model involves the use of the following eight propagation reactions.

$$\sim\!\sim M_1M_1\bullet + M_1 \xrightarrow{k_{111}} \sim\!\sim M_1M_1\bullet$$

$$\sim\!\sim M_1M_1\bullet + M_2 \xrightarrow{k_{112}} \sim\!\sim M_1M_2\bullet$$

$$\sim\!\sim M_1M_2\bullet + M_1 \xrightarrow{k_{121}} \sim\!\sim M_2M_1\bullet$$

$$\sim\!\sim M_1M_2\bullet + M_2 \xrightarrow{k_{122}} \sim\!\sim M_2M_2\bullet$$

$$\sim\!\sim M_2M_1\bullet + M_1 \xrightarrow{k_{211}} \sim\!\sim M_1M_1\bullet$$

$$\sim\!\sim M_2M_1\bullet + M_2 \xrightarrow{k_{212}} \sim\!\sim M_1M_2\bullet$$

$$\sim\!\sim M_2M_2\bullet + M_1 \xrightarrow{k_{221}} \sim\!\sim M_2M_1\bullet$$

$$\sim\!\sim M_2M_2\bullet + M_2 \xrightarrow{k_{222}} \sim\!\sim M_2M_2\bullet$$

The monomer reactivity ratios are $r_{11} = k_{111}/k_{112}$, $r_{12} = k_{122}/k_{121}$, $r_{21} = k_{211}/k_{212}$, and $r_{22} = k_{222}/k_{221}$.

With these four monomer reactivity ratios, the copolymer composition is represented as follows.

$$f = \frac{1+r_{21}F\left\{\left(r_{11}F+1\right)/\left(r_{21}F+1\right)\right\}}{1+(r_{12}/F)\left\{\left(r_{22}F+F\right)/\left(r_{12}F+F\right)\right\}} \qquad \text{(S2)}$$

where $F = [M_1]/[M_2]$ and $f = d[M_1]/d[M_2]$. F can be obtained from the experimental conditions and f can be obtained from the integral ratio of the ^{1}H NMR spectrum of the copolymer or the ratio of the monomer consumption rates. In this case, the r_{12} and r_{22} values can be regarded as zero due to the lack of the homopolymerization ability of monomer 1. Therefore, the above equation is reformulated as follows. The nonlinear least-squares method was used to calculate r_{12} and r_{22} values.[7,8)]

$$\frac{1-2f}{F(1-f)} = \frac{f}{F^2(1-f)}r_{22} - \frac{1}{r_{12}} \qquad \text{(S3)}$$

3. NMR spectra

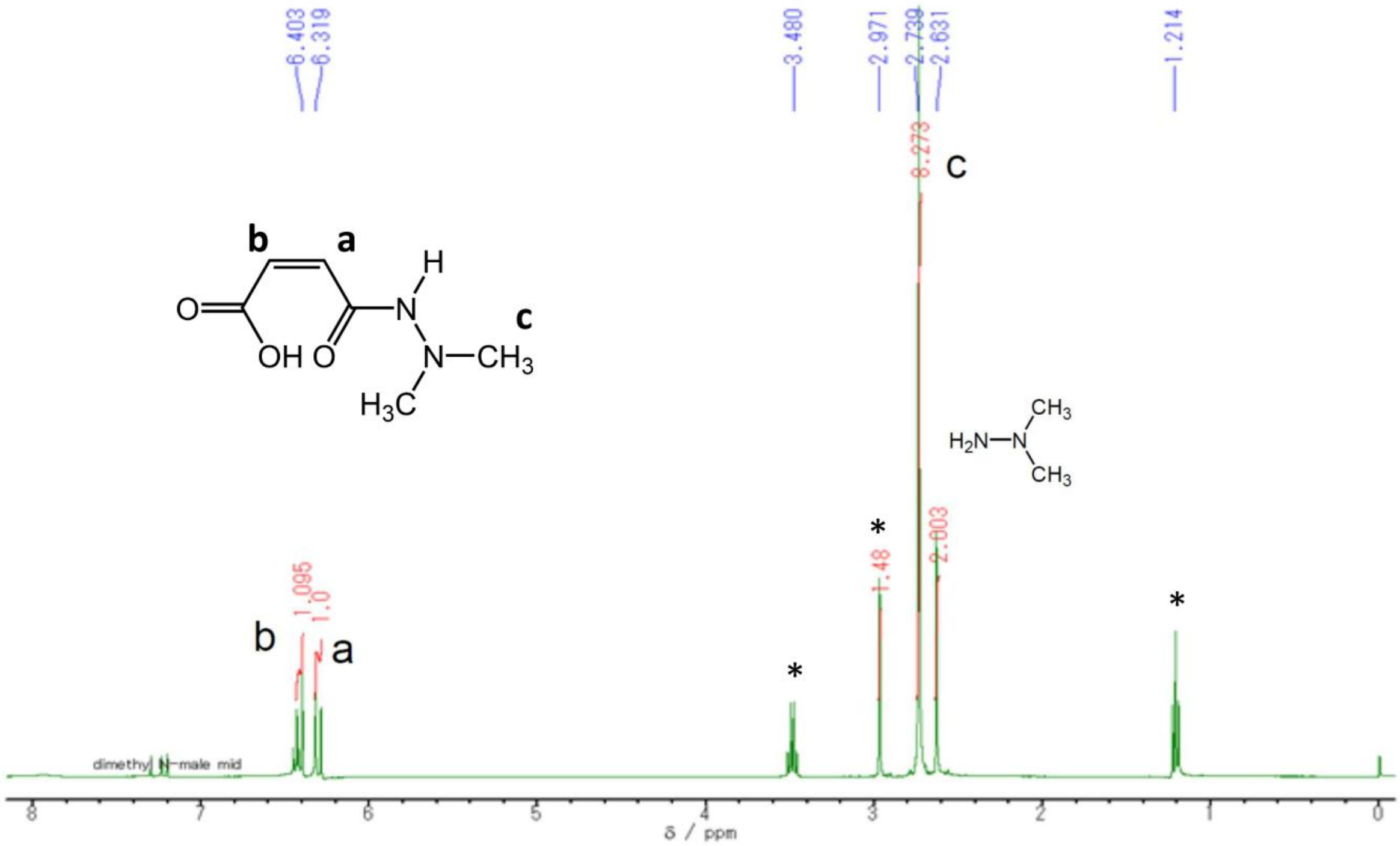

Figure S1. ^{1}H NMR spectrum of *N*-(*N′,N′*-dimethylamino)maleamic acid. Solvent $CDCl_3$.

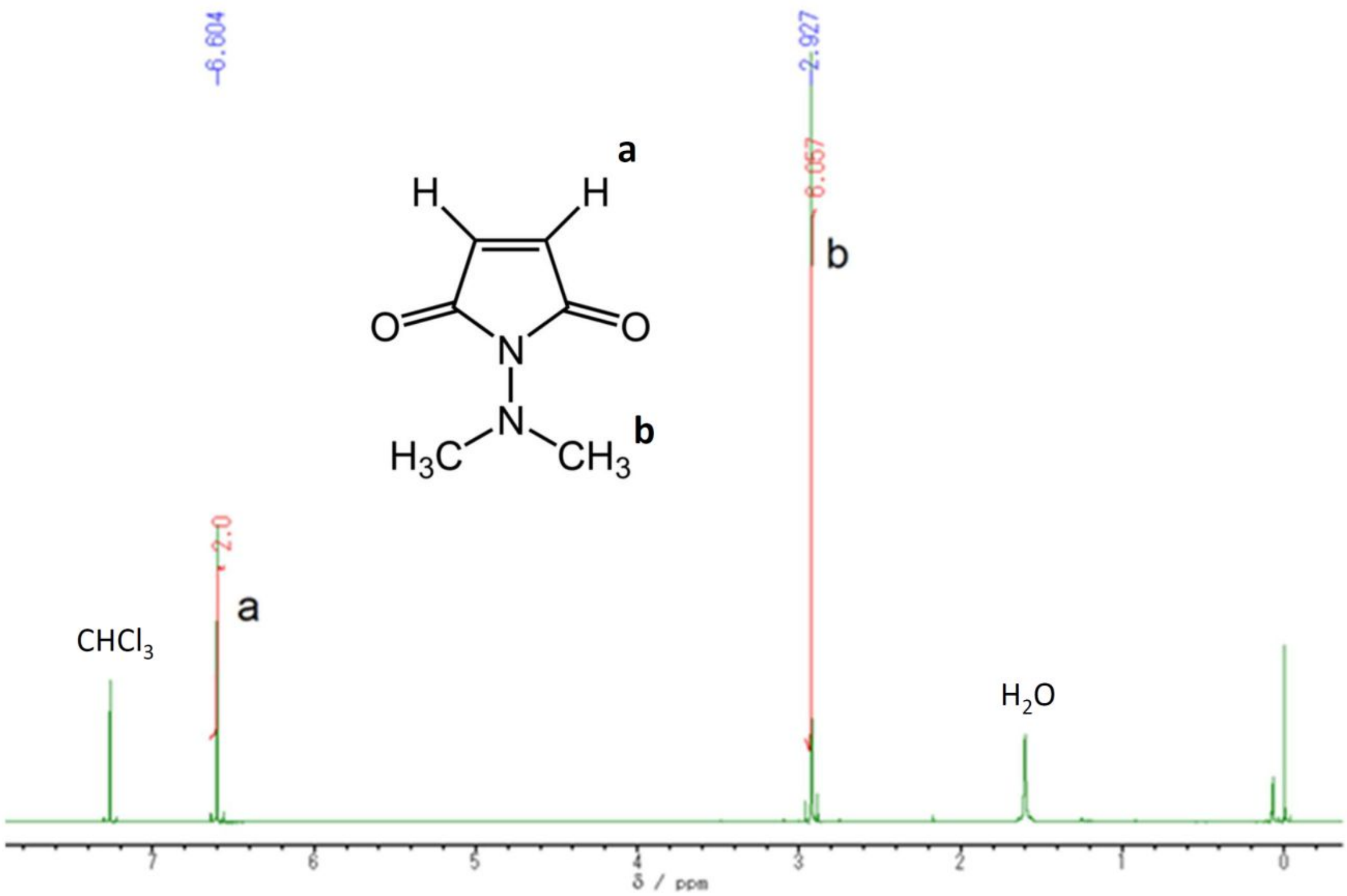

Figure S2. ^{1}H NMR spectrum of DMAMI. Solvent $CDCl_3$.

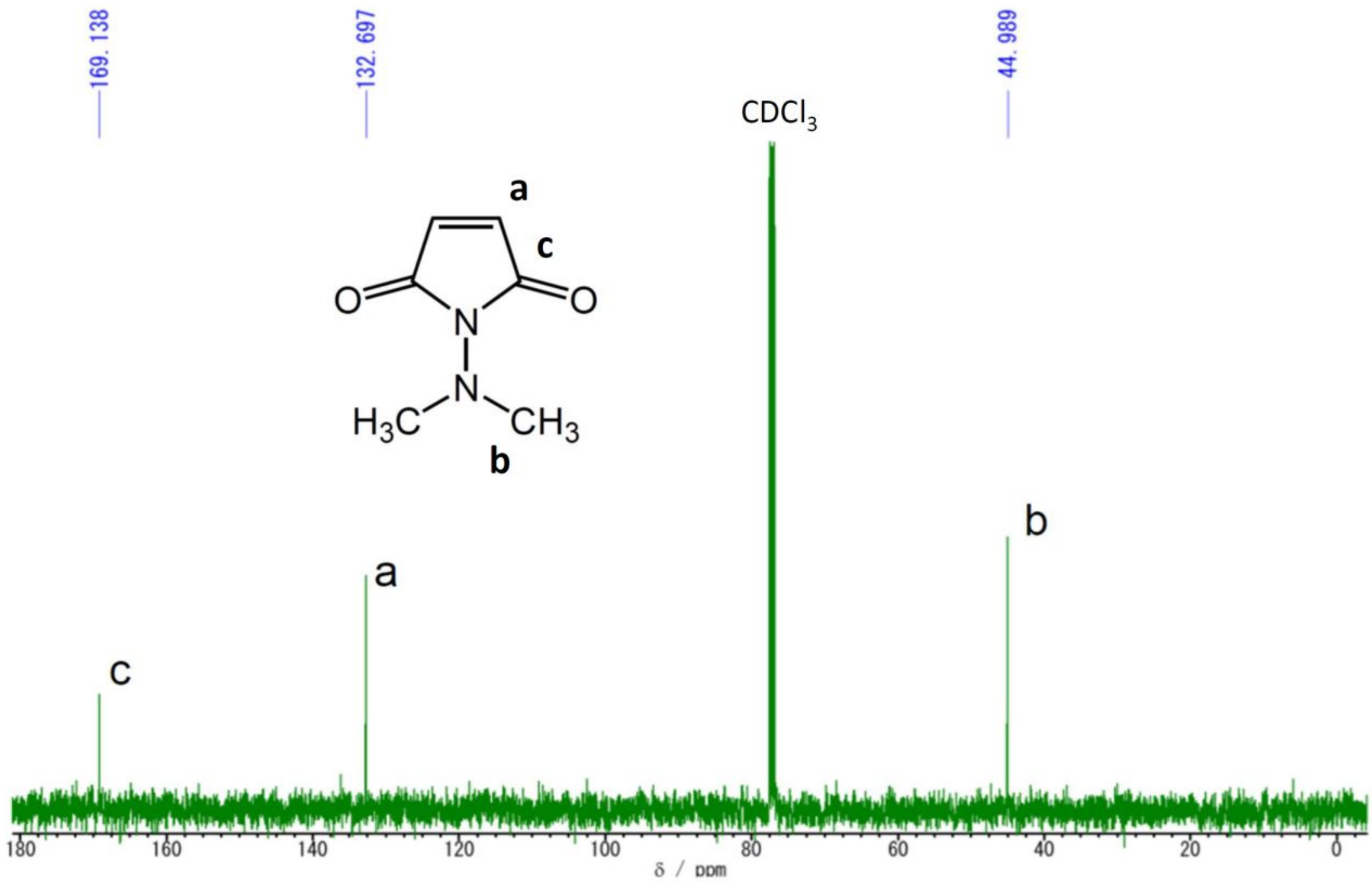

Figure S3. ^{13}C NMR spectrum of DMAMI. Solvent $CDCl_3$.

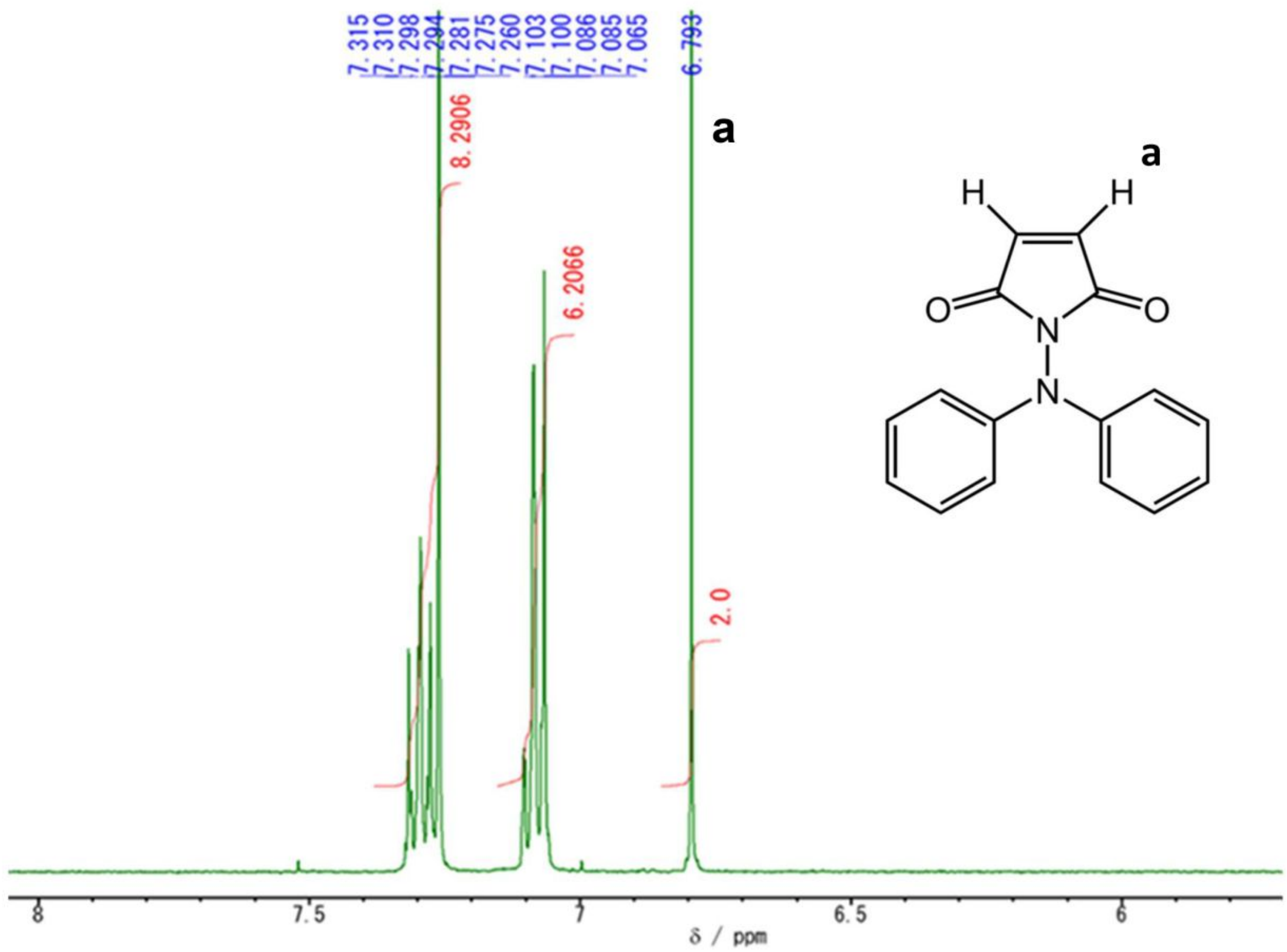

Figure S4. ^{1}H NMR spectrum of DPhAMI. Solvent $CDCl_3$.

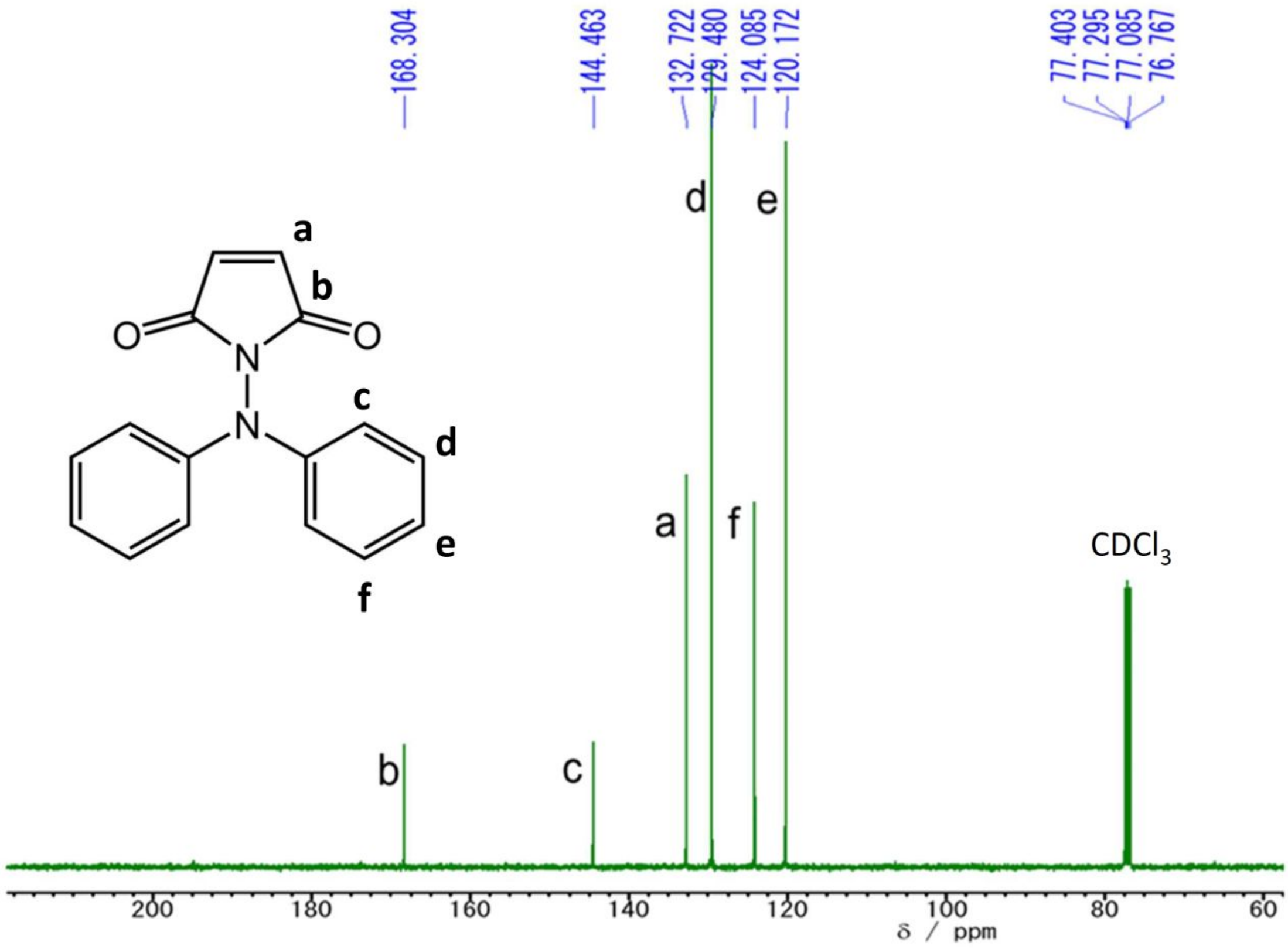

Figure S5. ^{13}C NMR spectrum of DPhAMI. Solvent $CDCl_3$.

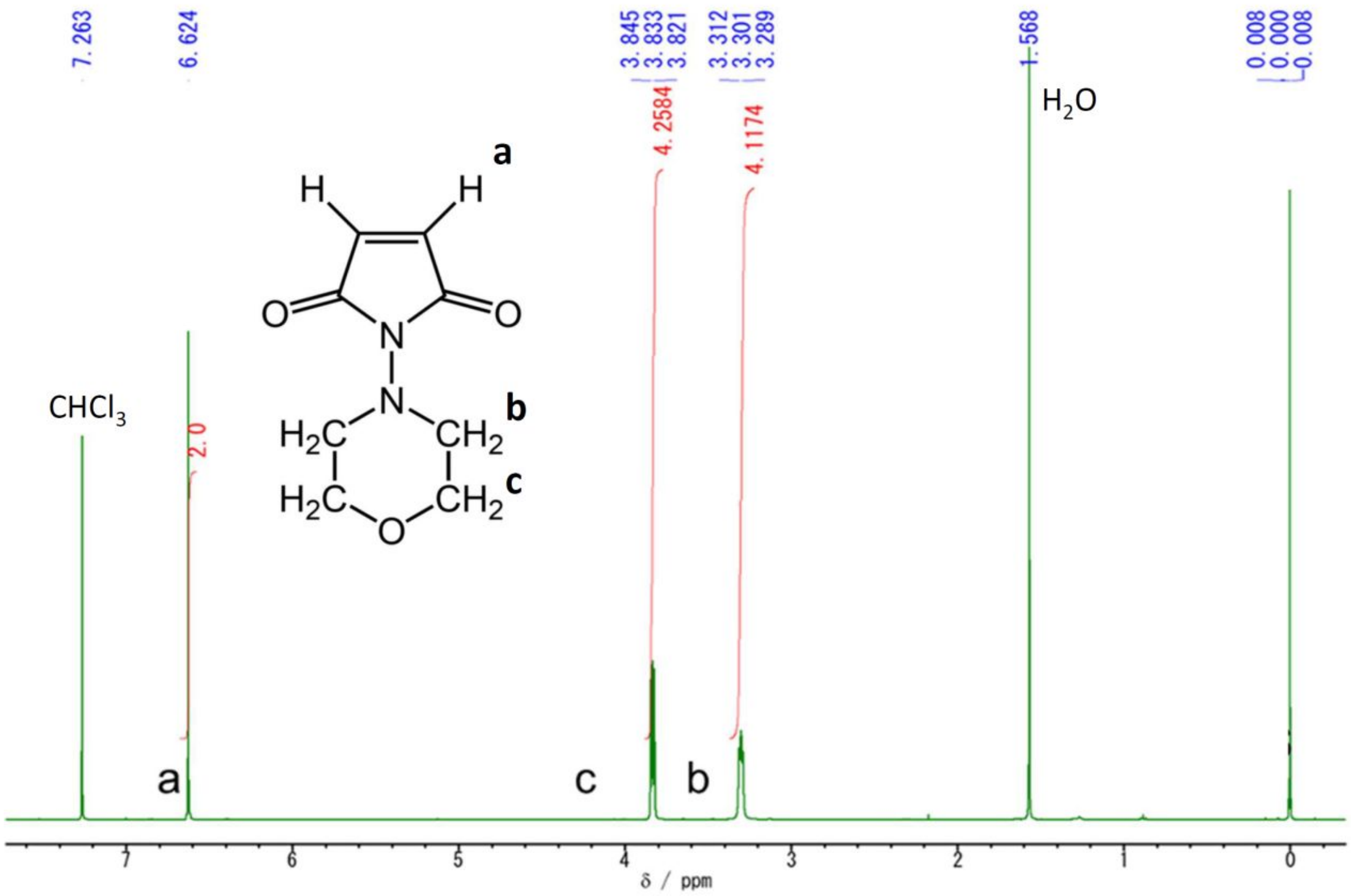

Figure S6. ^{1}H NMR spectrum of MorMI. Solvent $CDCl_3$.

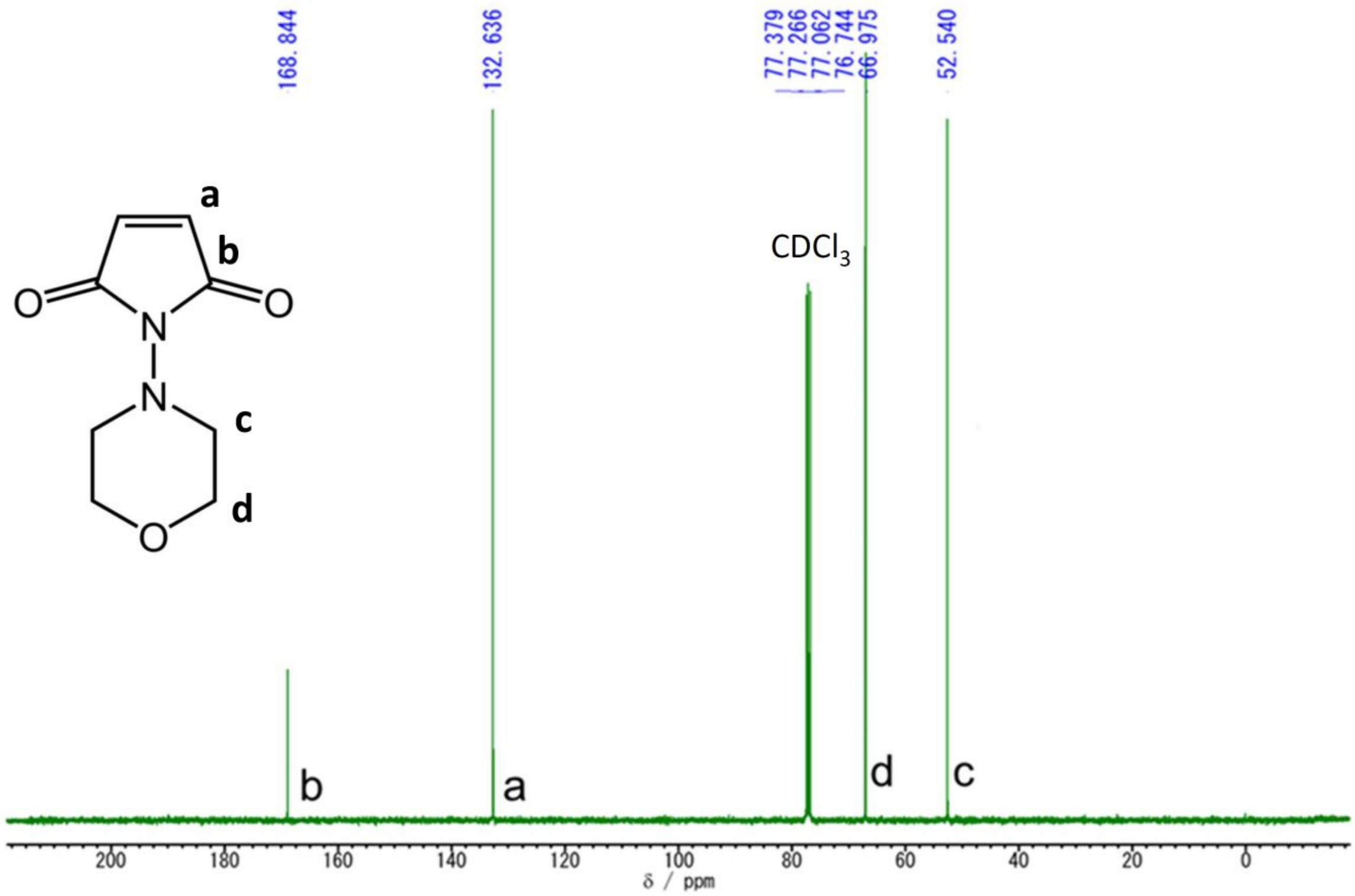

Figure S7. ^{13}C NMR spectrum of MorMI. Solvent $CDCl_3$.

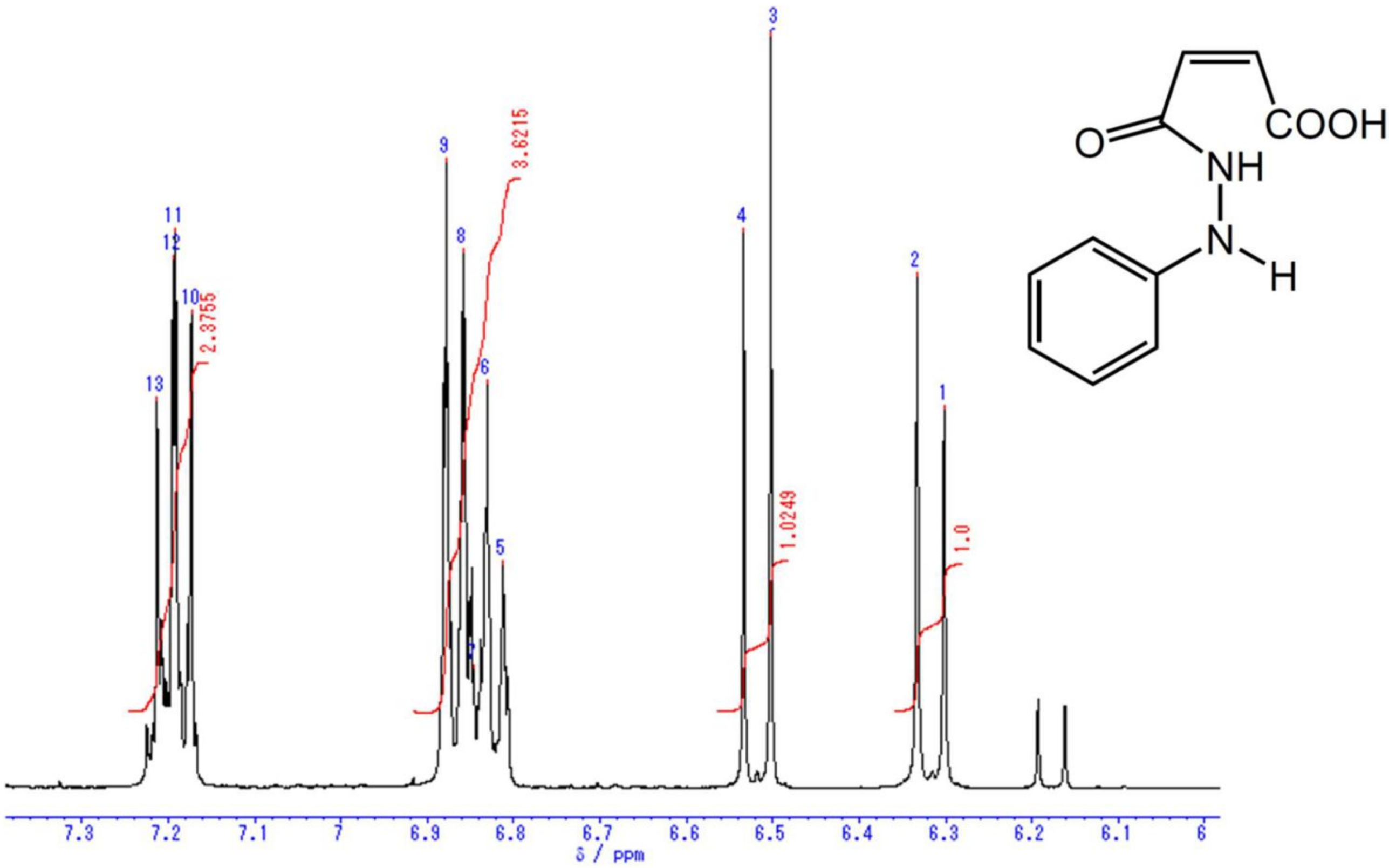

Figure S8. ^{1}H NMR spectrum of *N*-(*N′*-phenylamino)maleamic acid. Solvent $CDCl_3$.

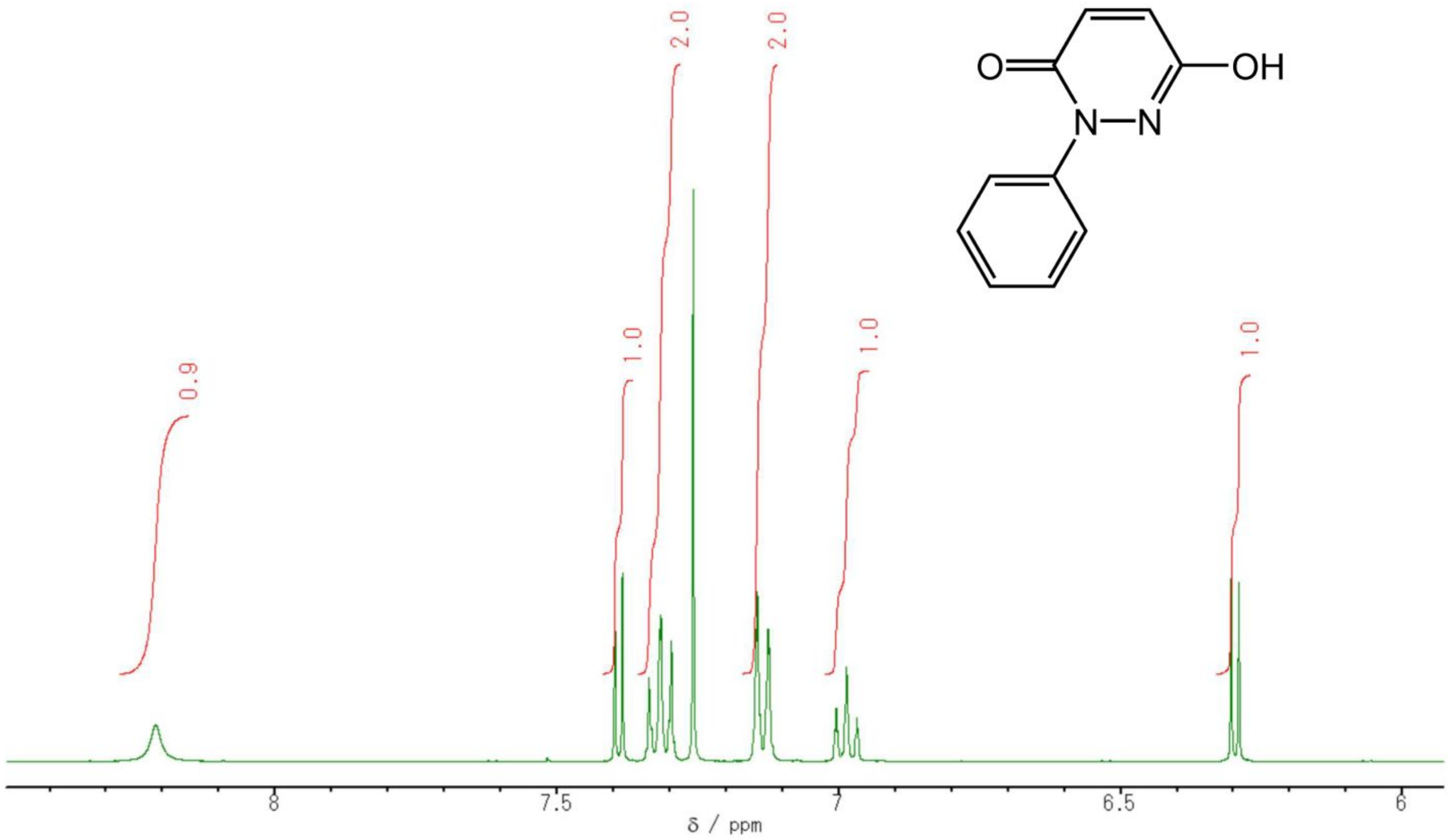

Figure S9. ^{1}H NMR spectrum of Product B. Solvent $CDCl_3$.

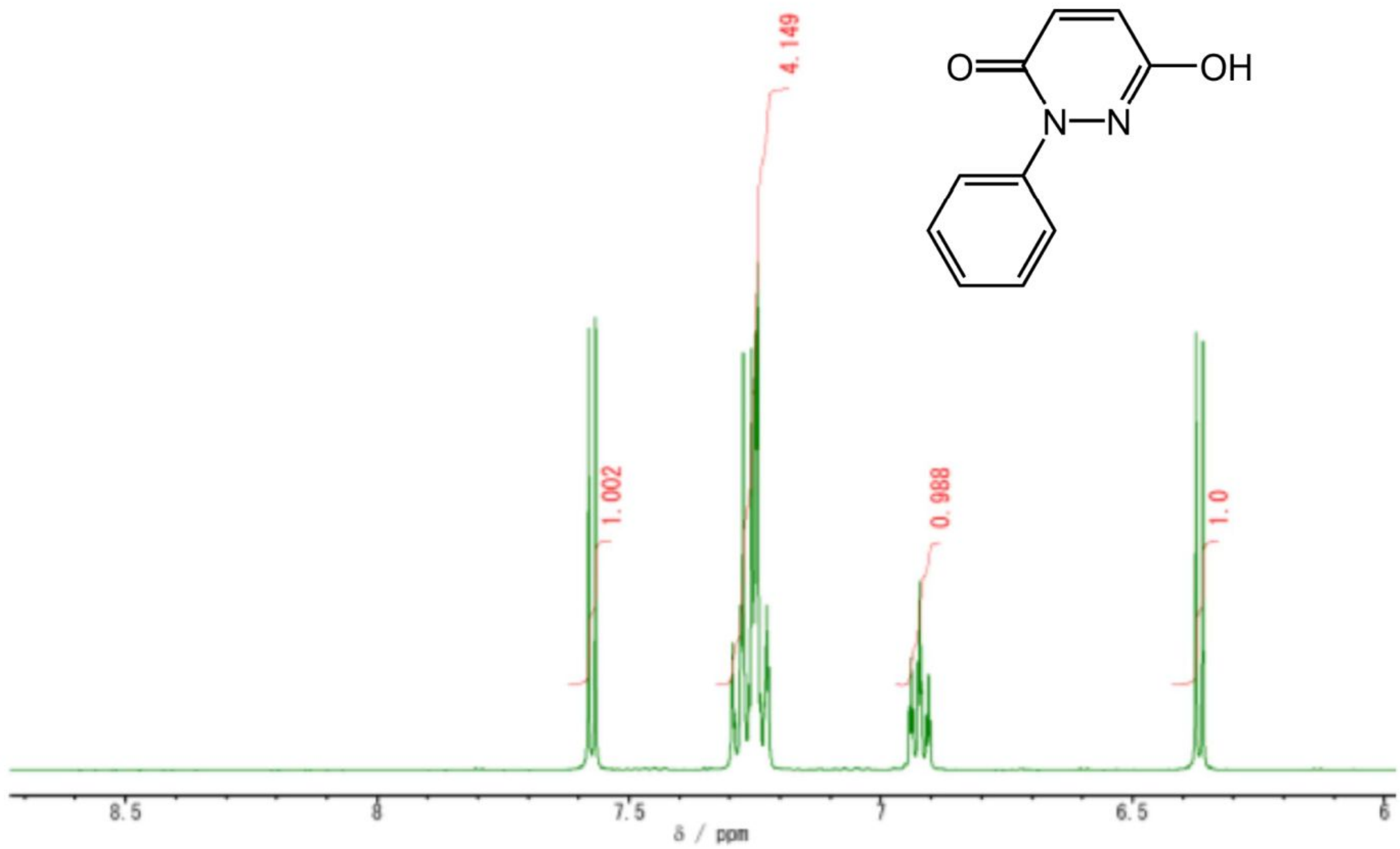

Figure S10. ^{1}H NMR spectrum of Product B. Solvent CD_3OD.

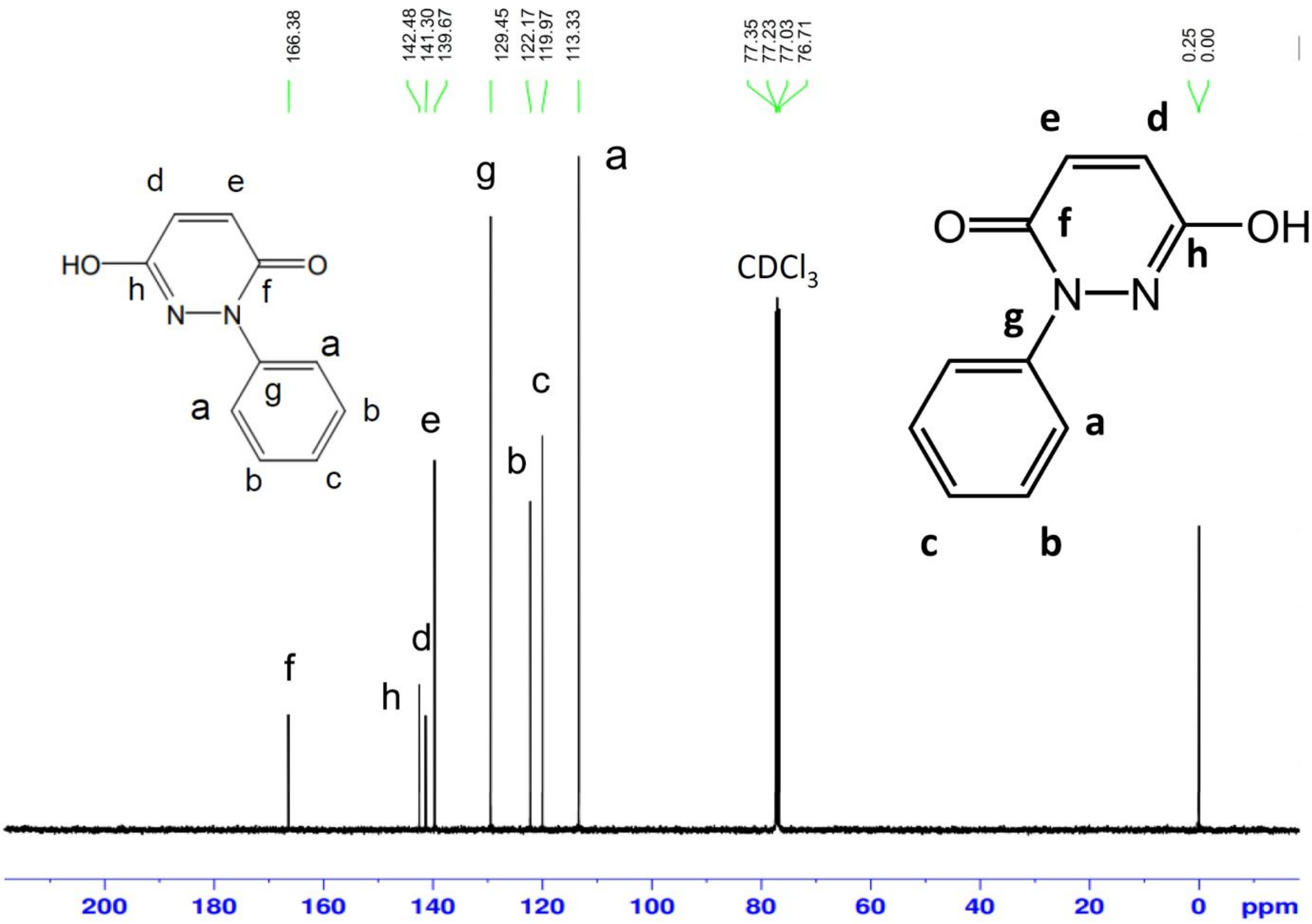

Figure S11. ^{13}C NMR spectrum of Product B. Solvent $CDCl_3$.

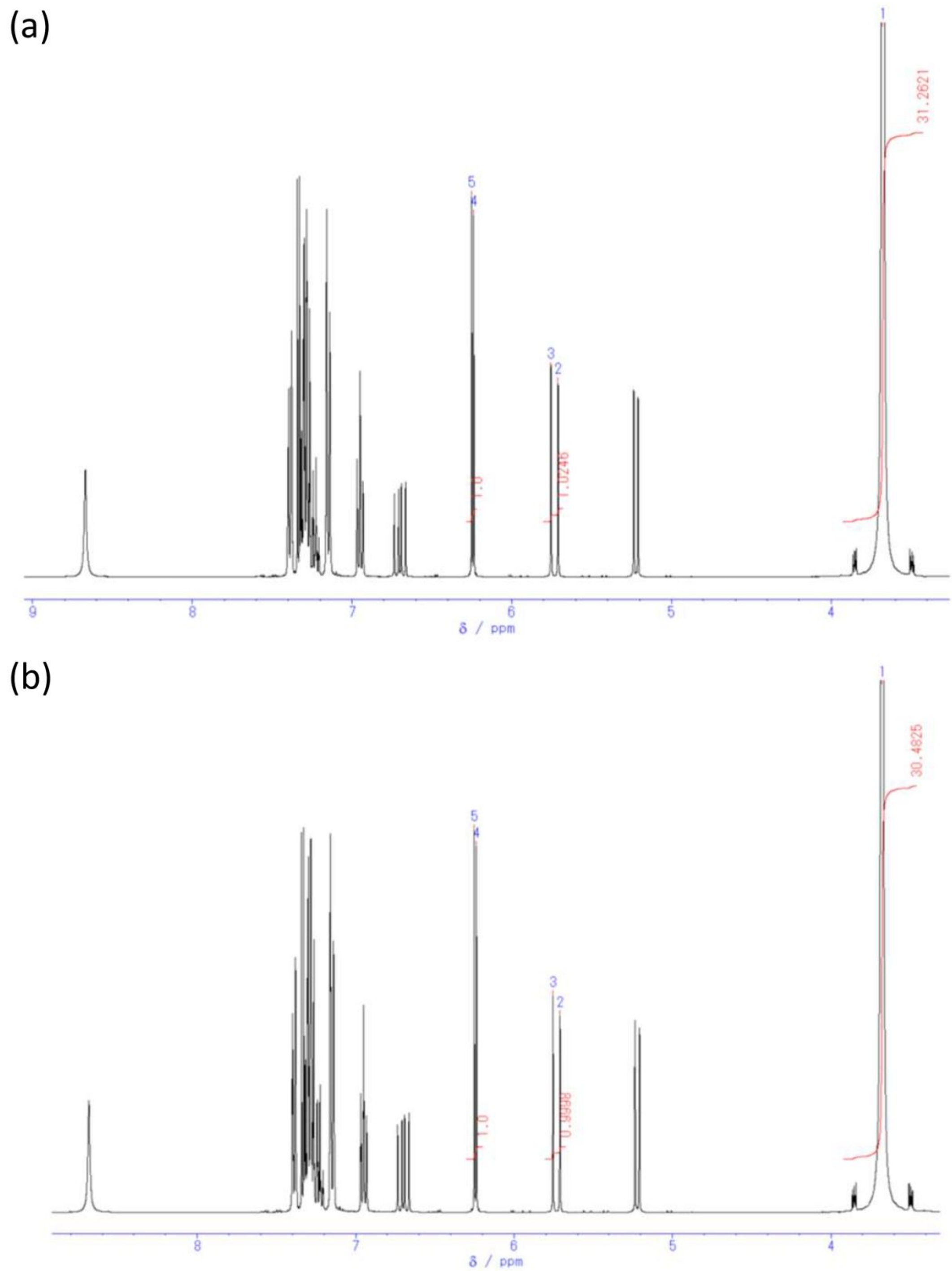

Figure S12. ^{1}H NMR spectra of the polymerization system of Product B with St in $CDCl_3$ in the presence of AIBN. Heating at 60 C for (a) 0 h and (b) 5 h.

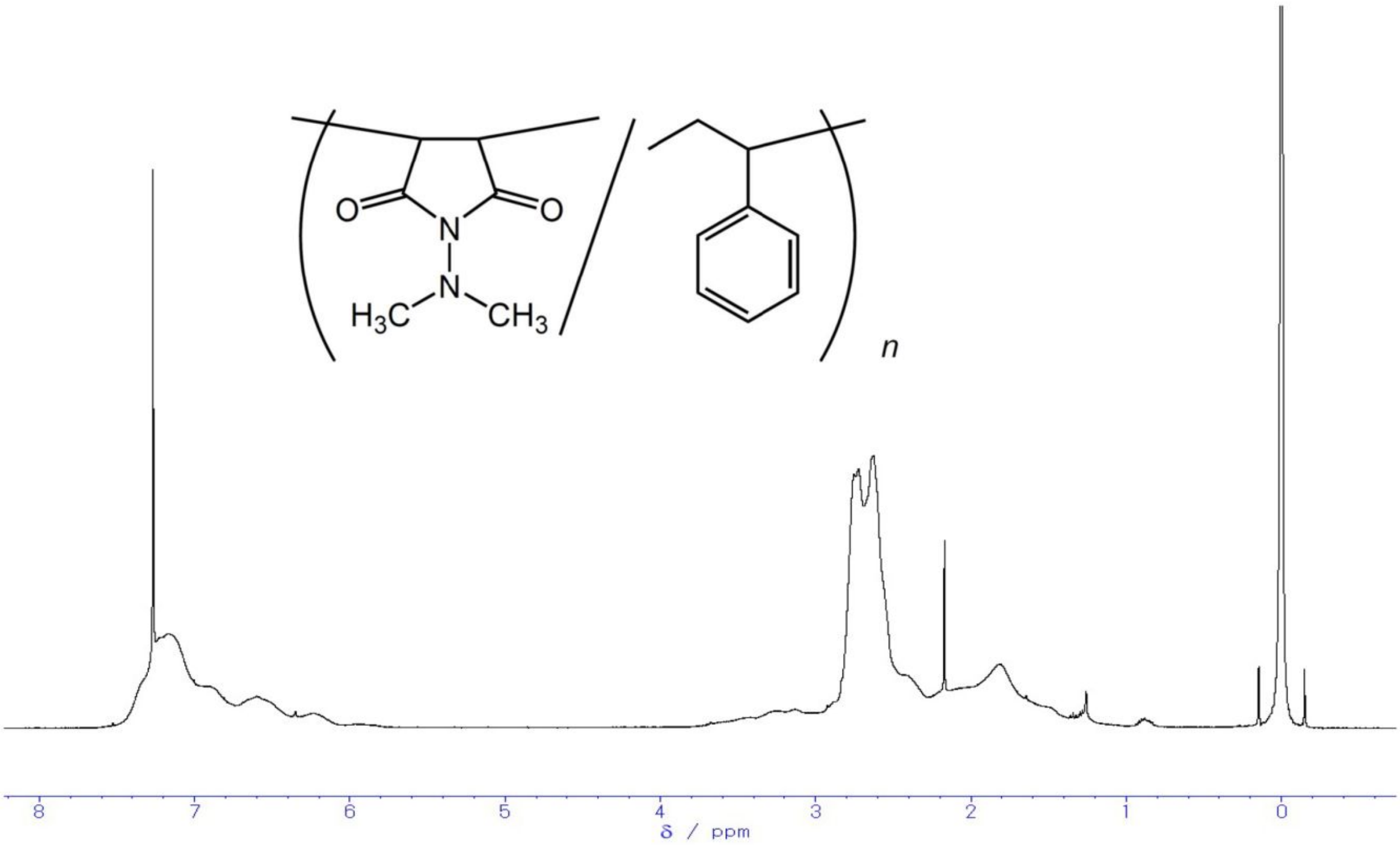

Figure S13. ^{1}H NMR spectrum of the copolymer of DMAMI and St. DMAMI content 50.3 mol% in the copolymer. Solvent $CDCl_3$.

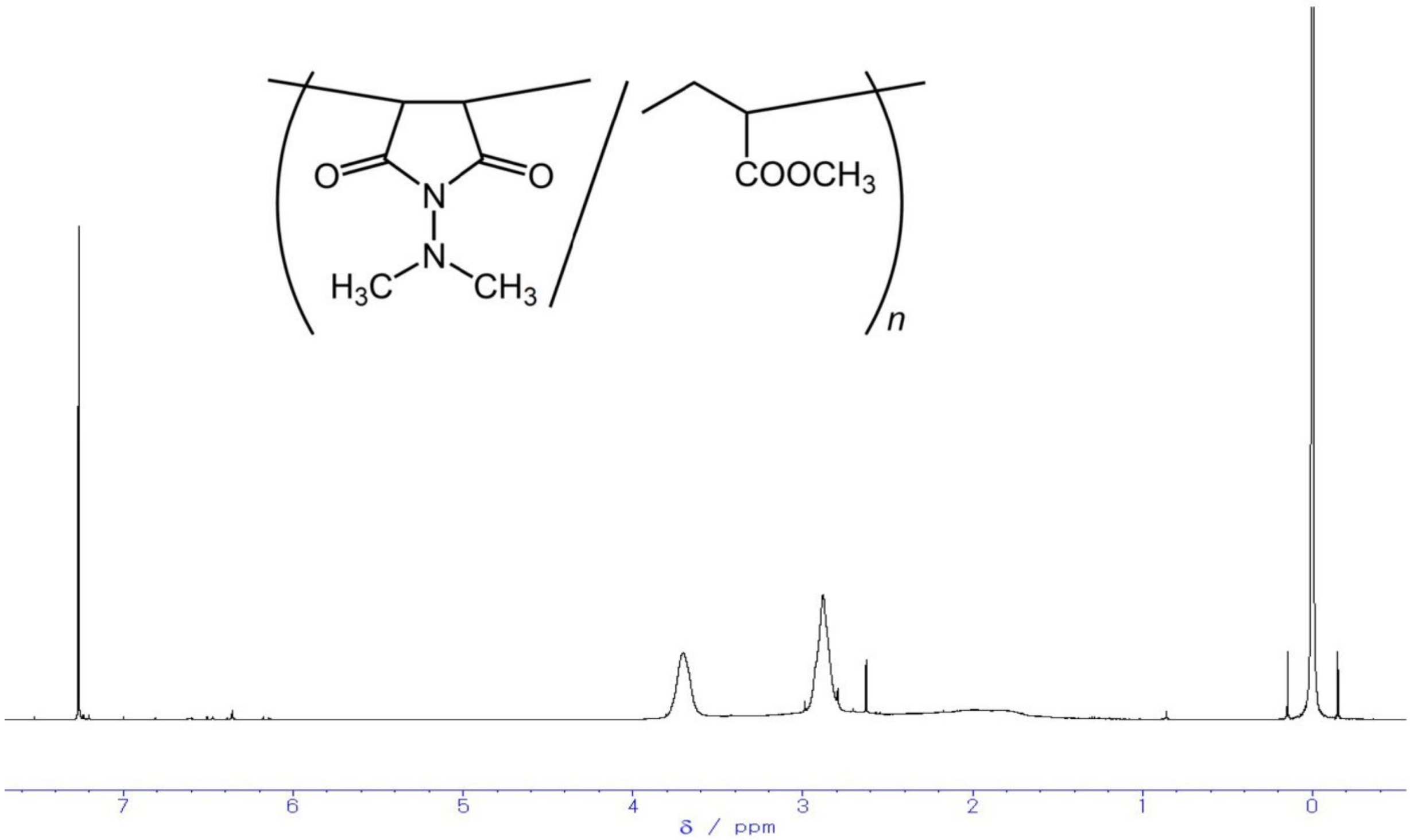

Figure S14. ^{1}H NMR spectrum of the copolymer of DMAMI and MA. DMAMI content 43.8 mol% in the copolymer. Solvent $CDCl_3$.

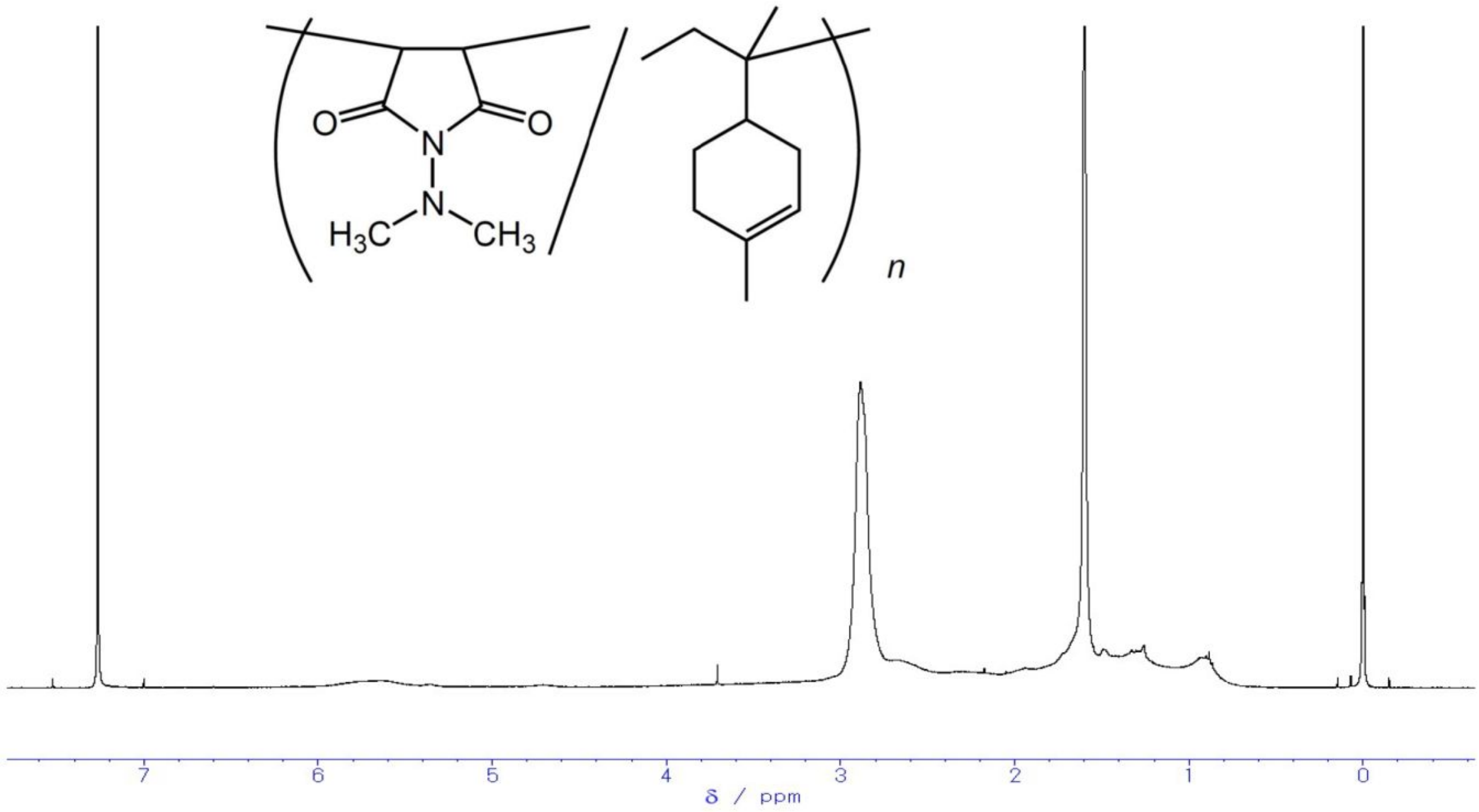

Figure S15. ^{1}H NMR spectrum of the copolymer of DMAMI and Lim. DMAMI content 65.3 mol% in the copolymer. Solvent $CDCl_3$.

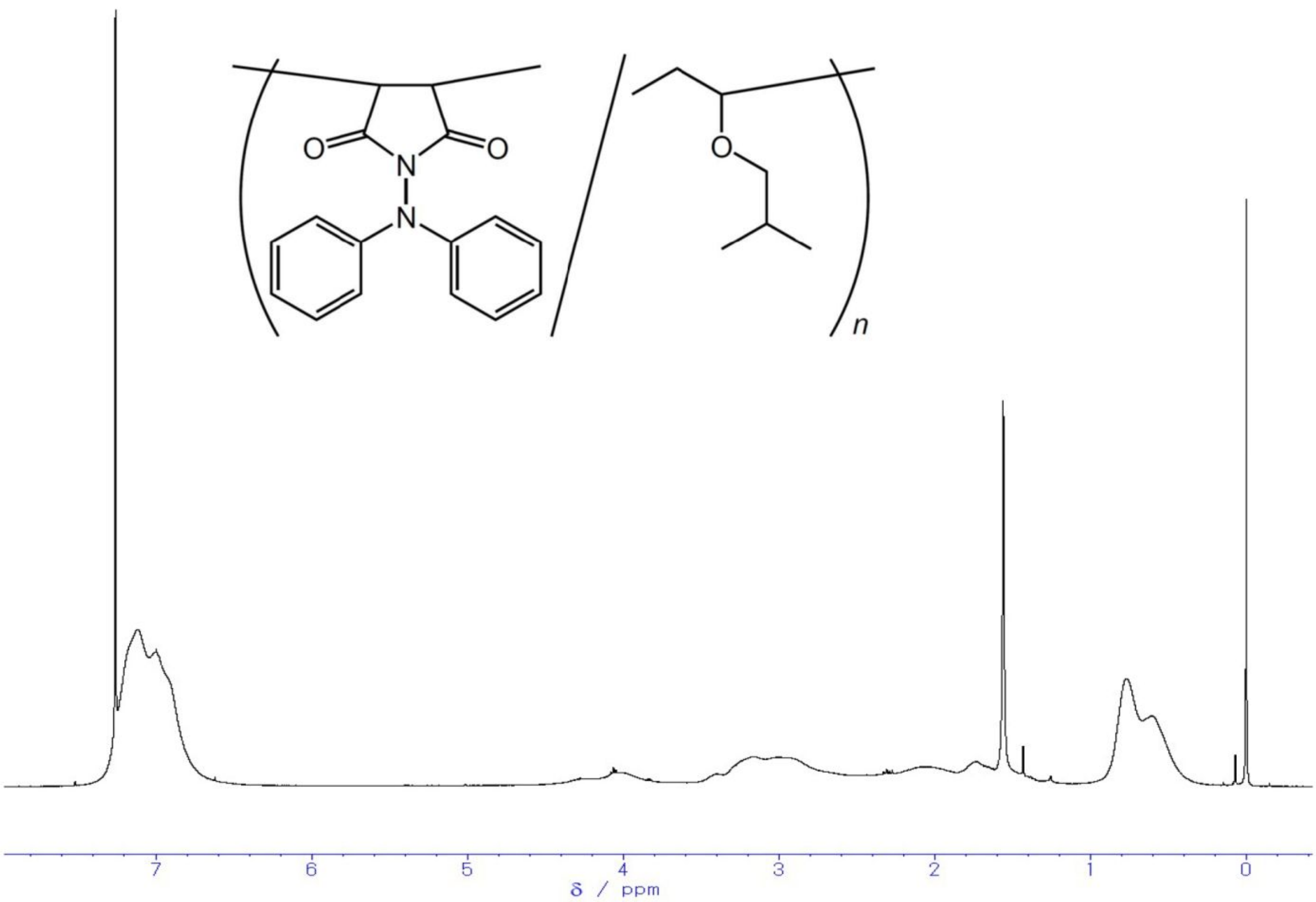

Figure S16. ^{1}H NMR spectrum of the copolymer of DPhAMI and IBVE. DPhAMI content 51.4 mol% in the copolymer. Solvent $CDCl_3$.

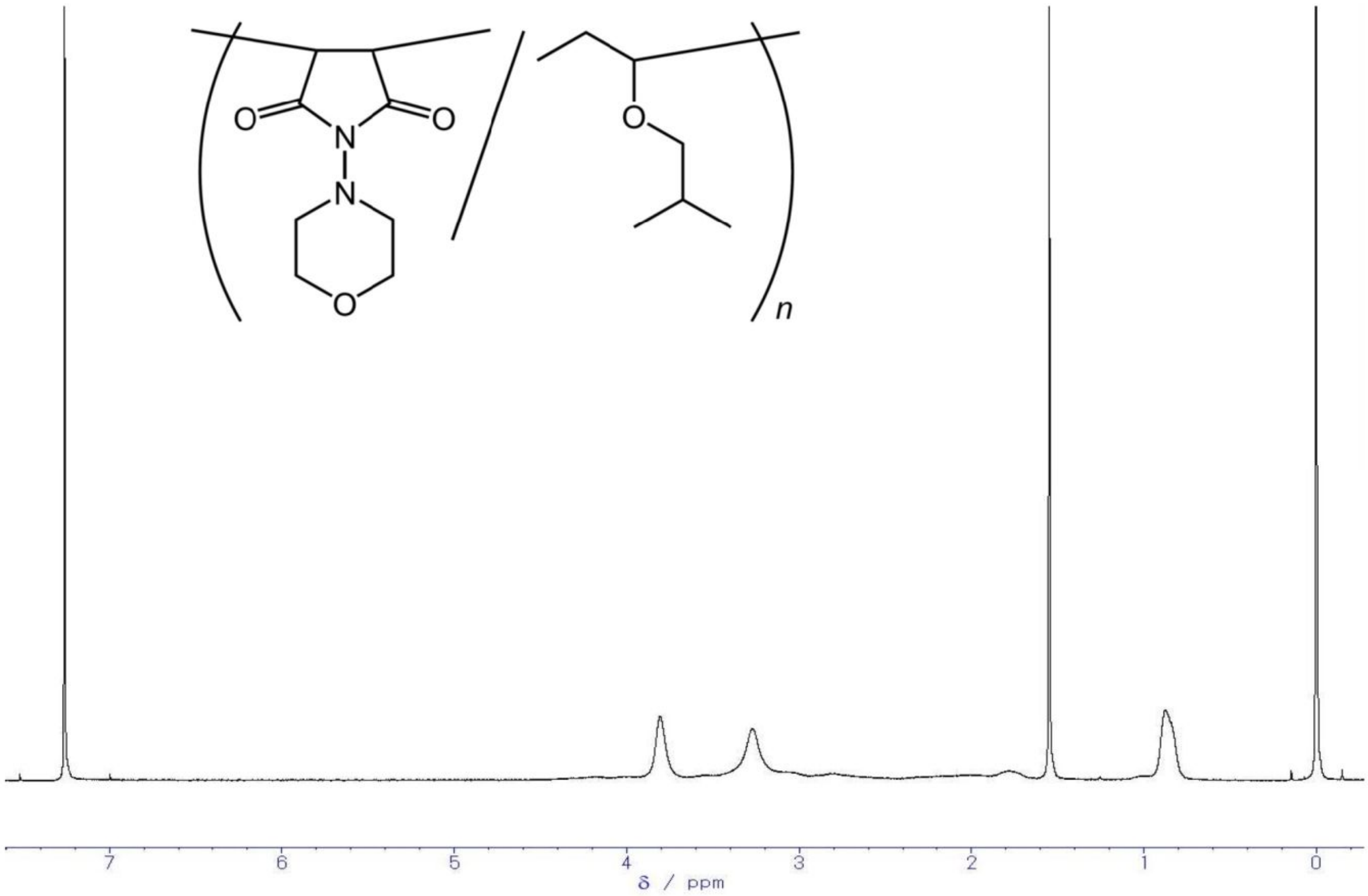

Figure S17. ^{1}H NMR spectrum of the copolymer of MorMI and IBVE. MorMI content 50.6 mol% in the copolymer. Solvent $CDCl_3$.

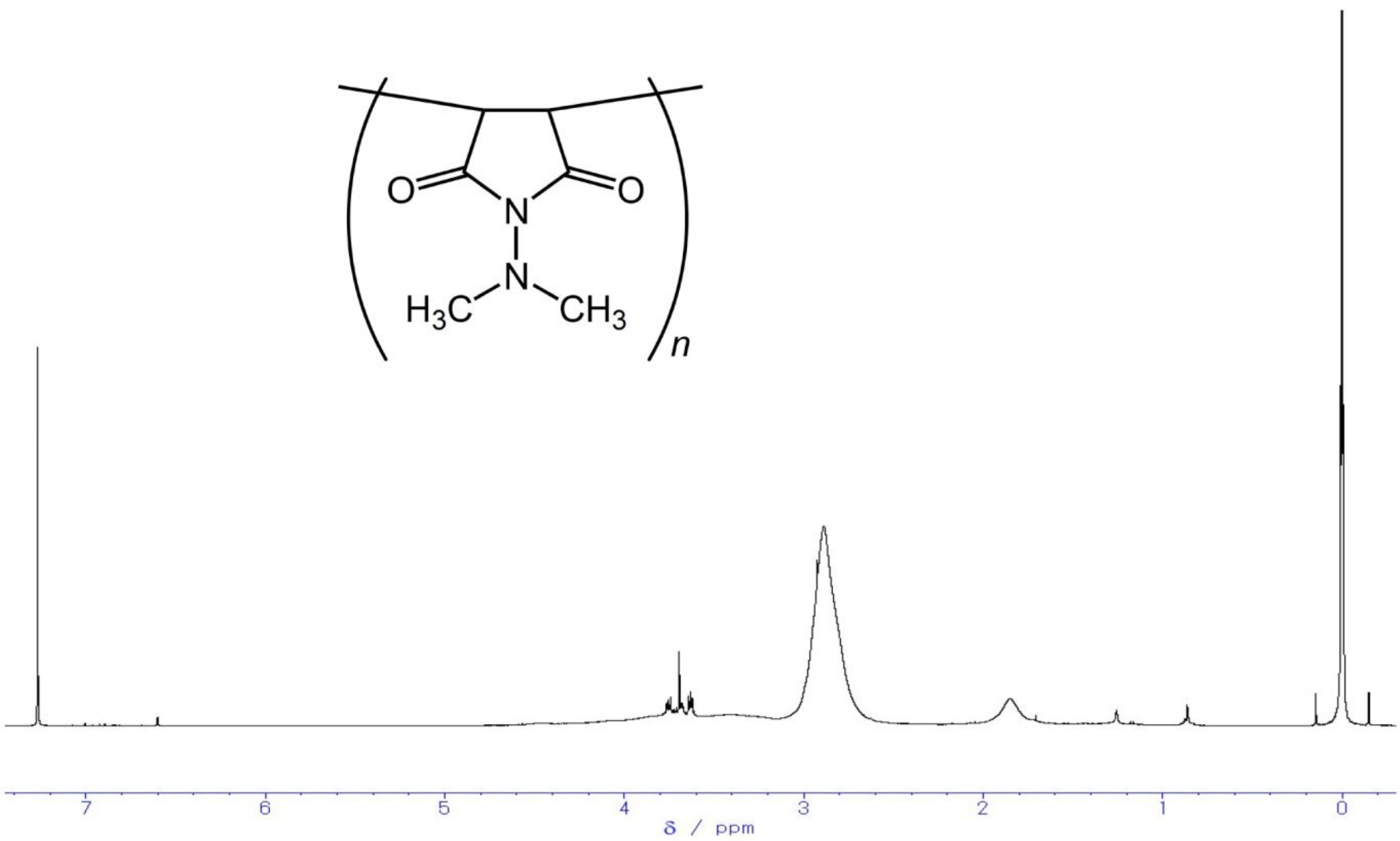

Figure S18. ^{1}H NMR spectra of the homopolymer of DMAMI. Solvent $CDCl_3$.

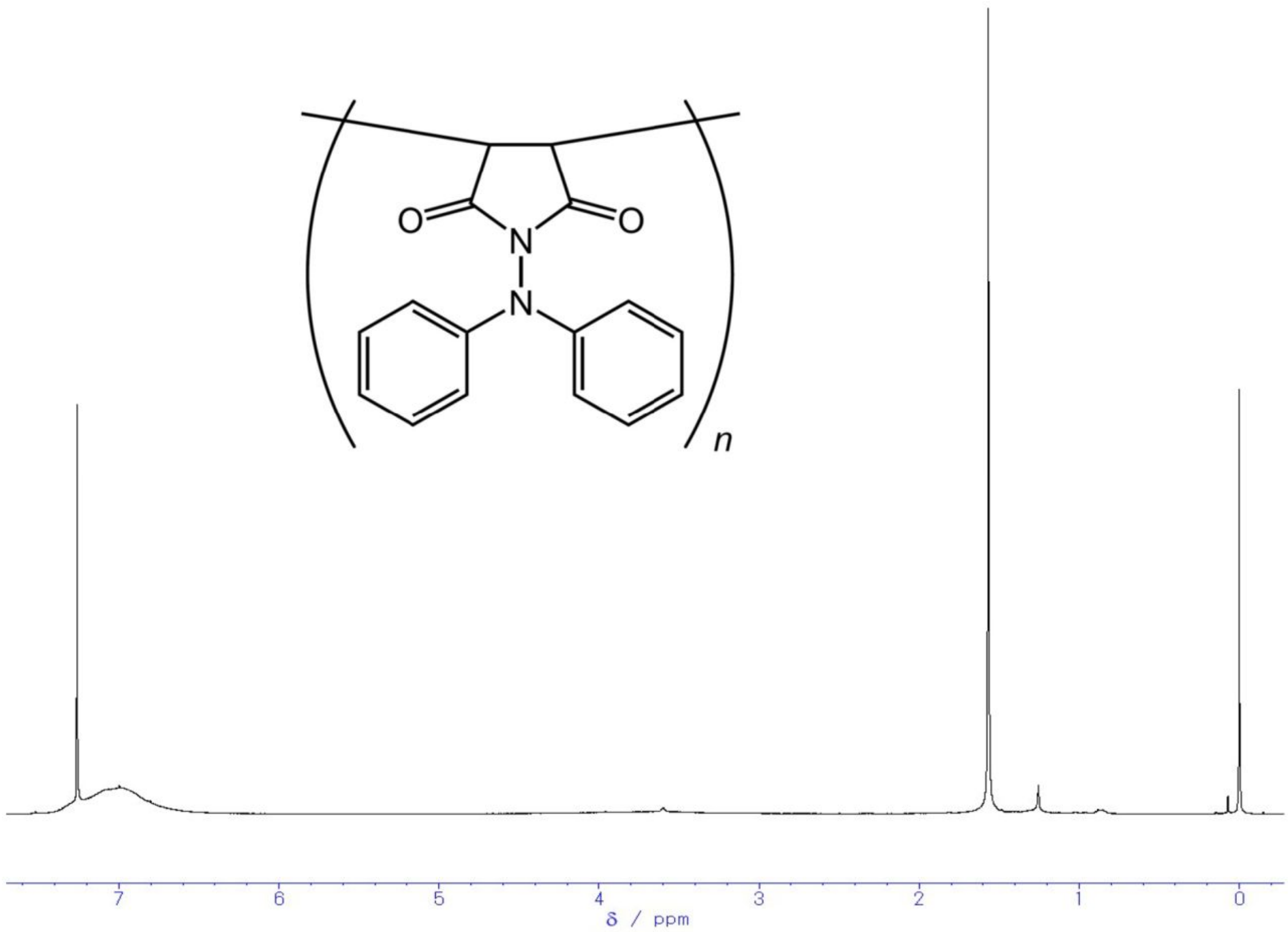

Figure S19. ^{1}H NMR spectra of the homopolymer of DPhAMI. Solvent $CDCl_3$.

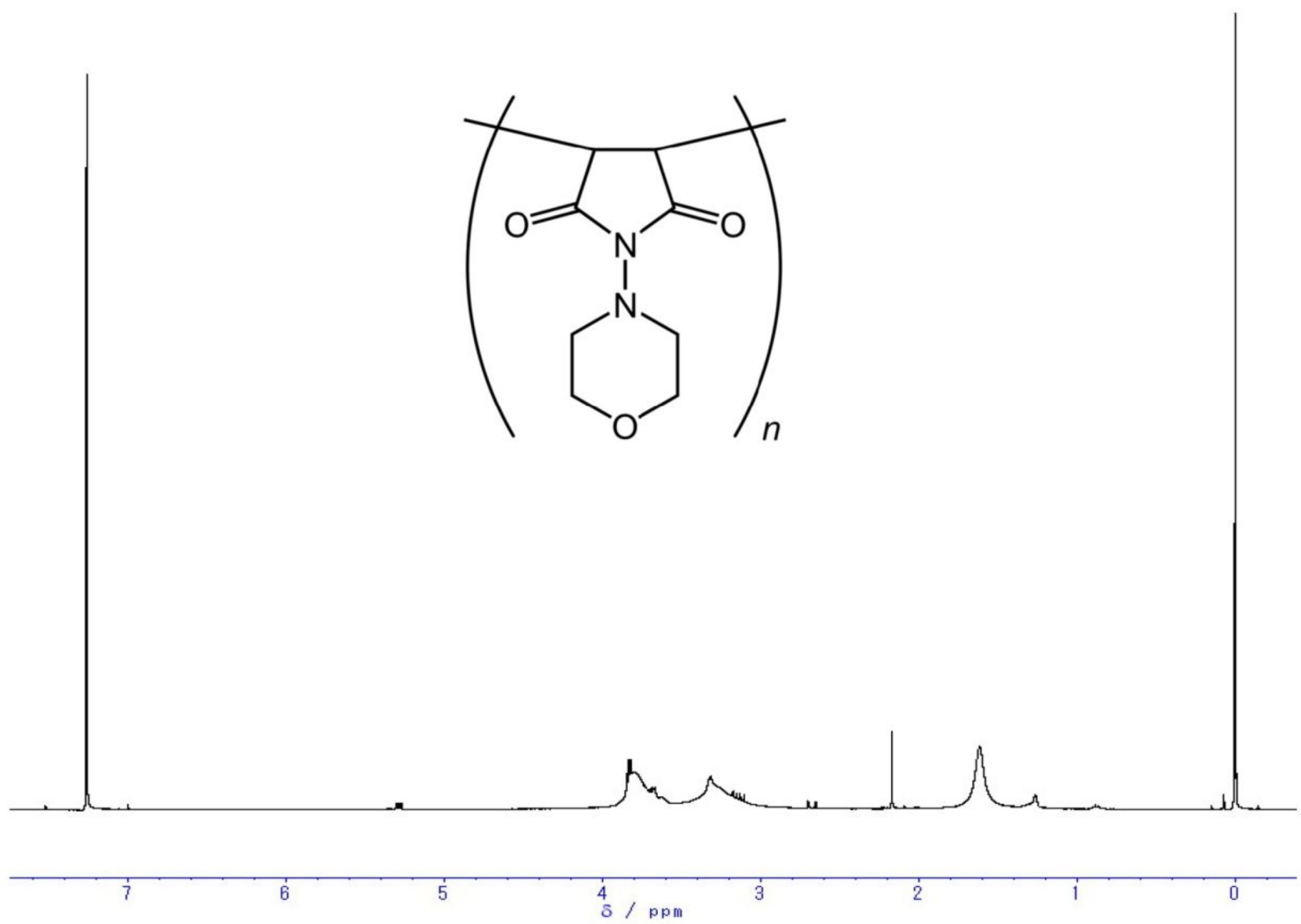

Figure S20. ^{1}H NMR spectra of the homopolymer of MorMI. Solvent $CDCl_3$.

4. UV-vis spectra

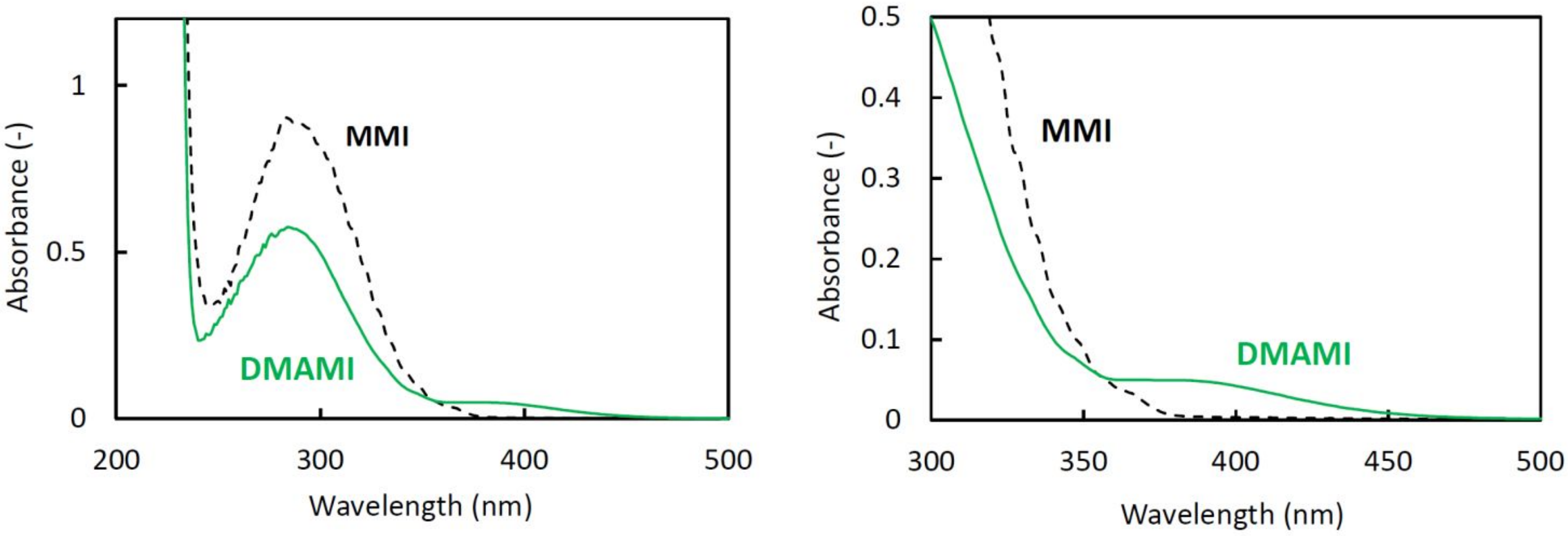

Figure S21. UV-vis spectra of DMAMI and MMI in *n*-hexane. λ_{max} = 284 nm, ε_{max} = 612 L/mol·cm for DMAMI; λ_{max} = 283 nm, ε_{max} = 940 L/mol·cm for MMI,

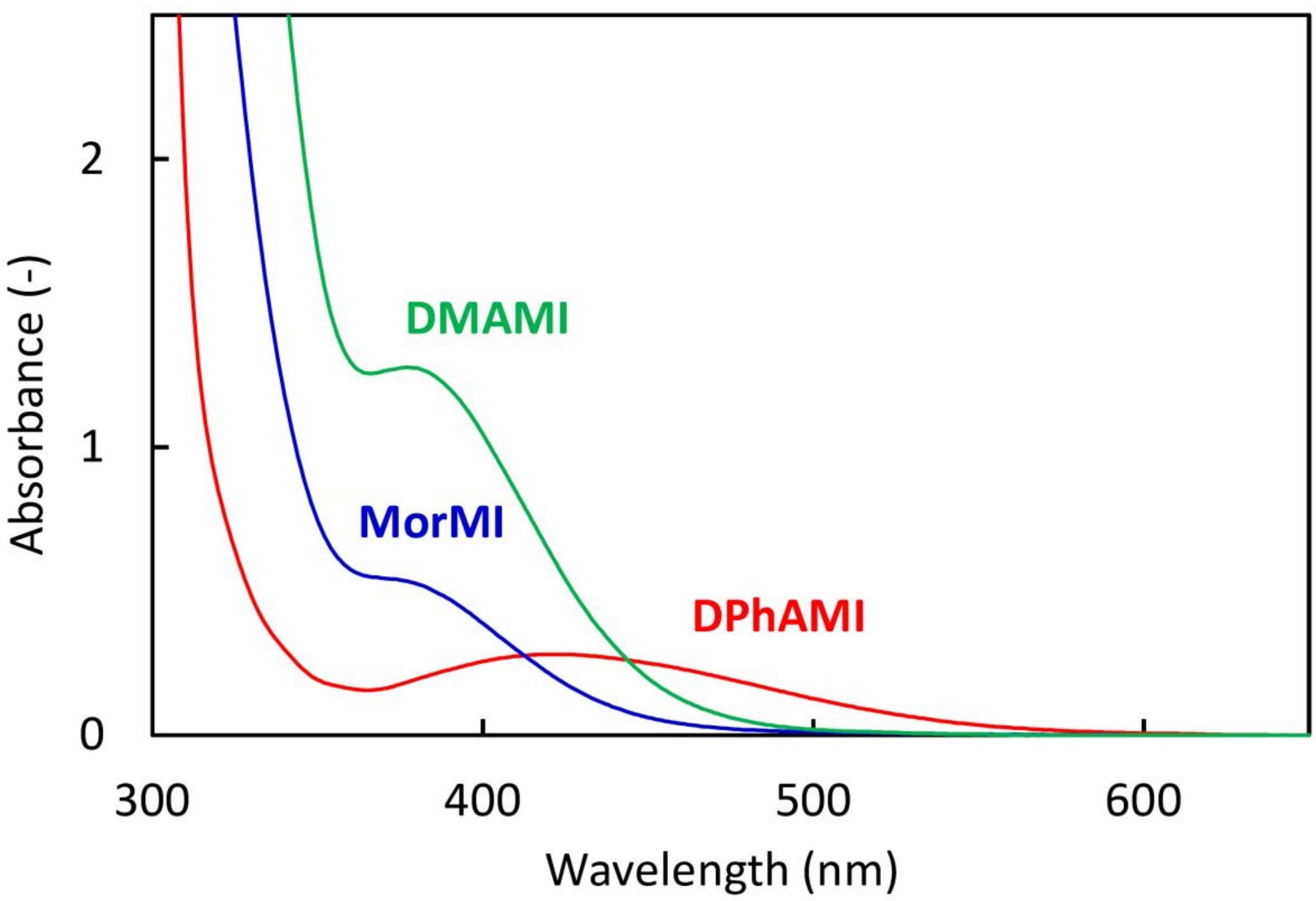

Figure S22. UV-vis spectra of DMAMI, DPhAMI, and MorMI.

5. IR spectra

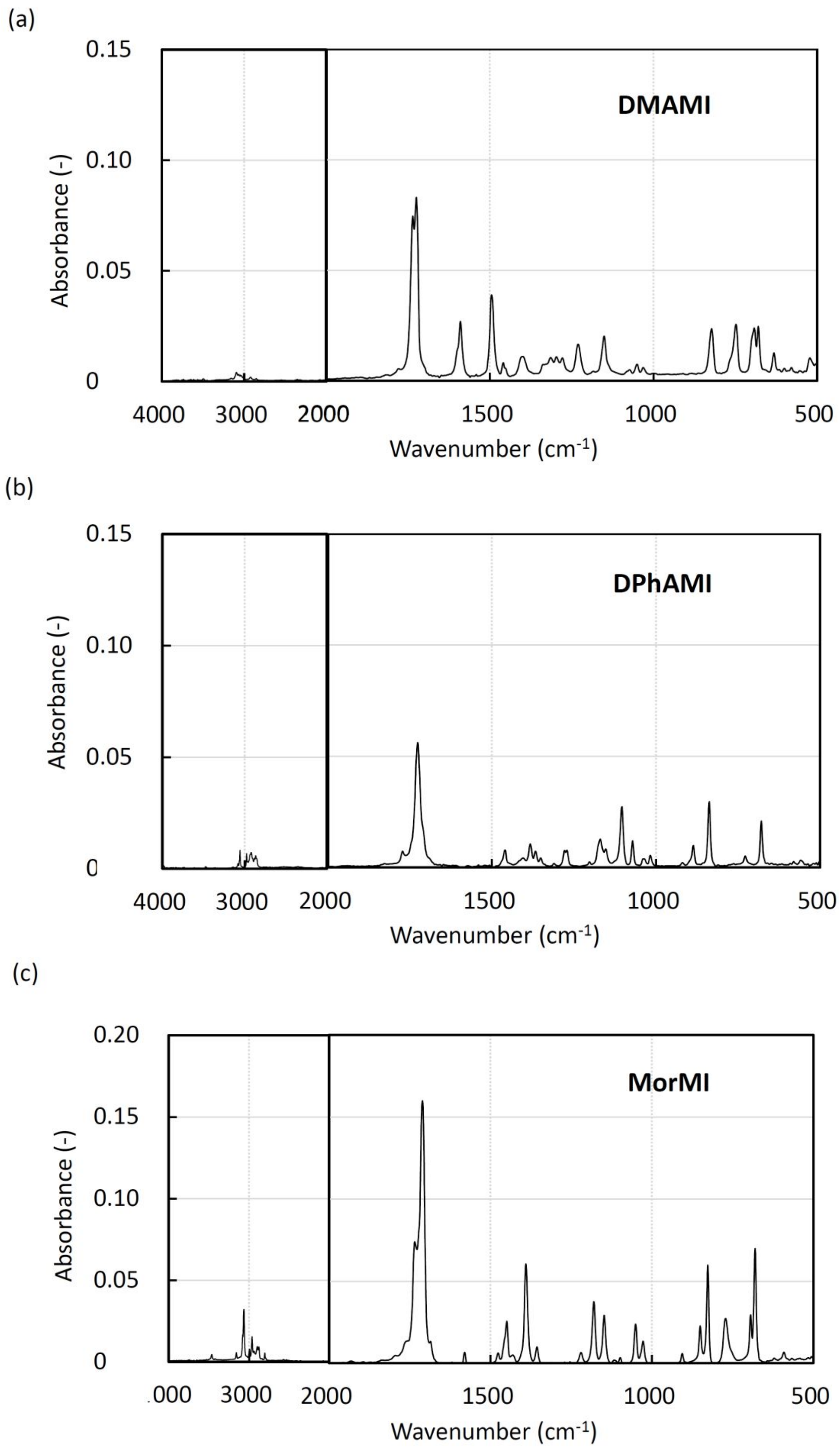

Figure S23. IR spectra of (a) DMAMI, (b) DPhAMI, and (c) MorMI.

6. Oxidative decomposition of the AMI copolymer

(a)

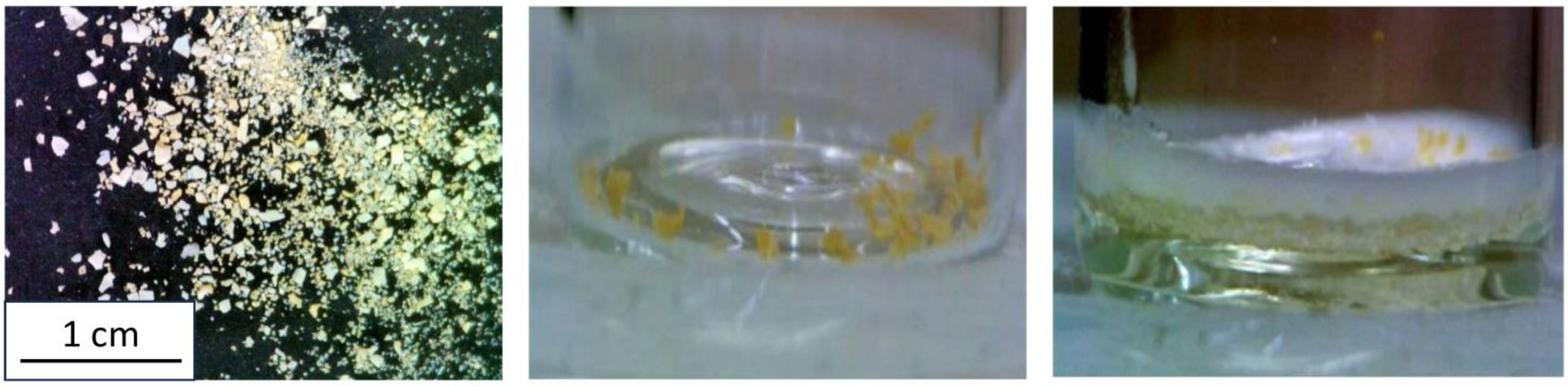

(b)

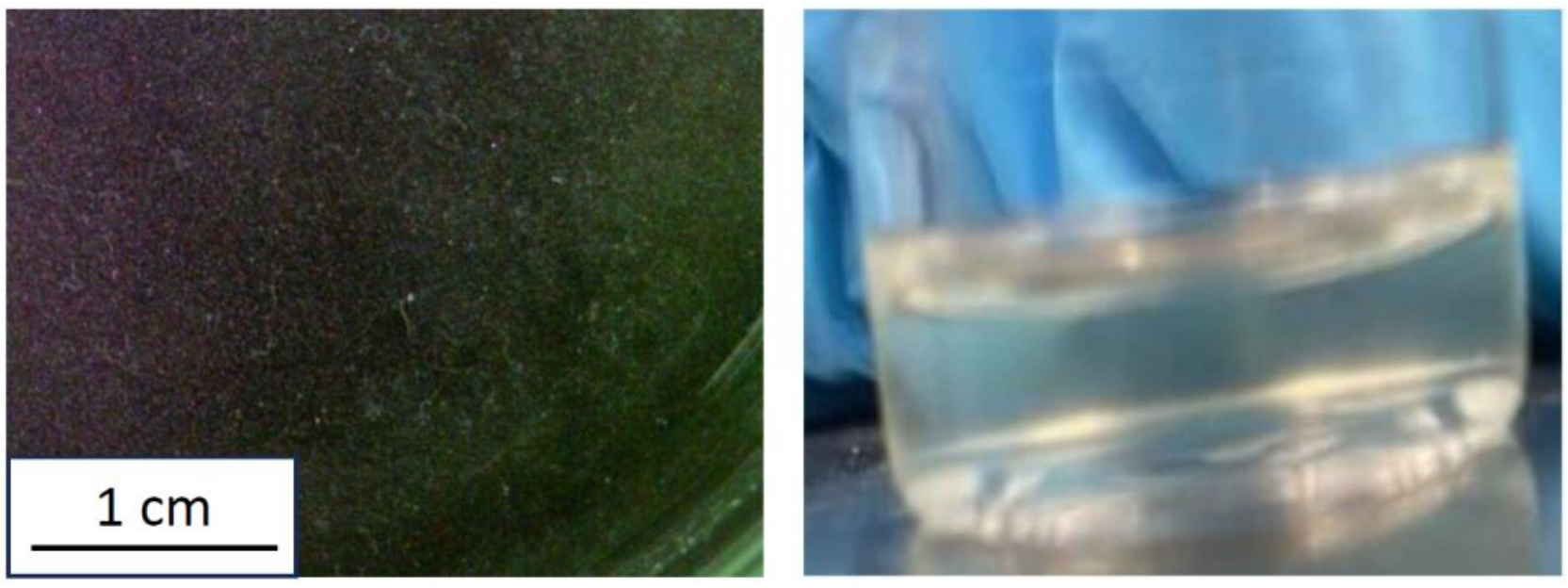

Figure S24. Photographs for oxidative decomposition[9,10)] of MorMI/IBVE copolymer in a sodium hypochlorite aqueous solution. (a) Appearance of the copolymer in the solid state and just after the addition of sodium hypochlorite aqueous solution and (b) after 2 h at room temperature.

Video S1

Change in the appearance for oxidative decomposition of MorMI/IBVE copolymer just after the addition of sodium hypochlorite aqueous solution.

7. Monomer reactivity ratio reported in the literature

Table S1. Monomer Reactivity Ratios for Radical Copolymerization of Maleimides (M_1) with Vinyl Monomers (M_2) Reported in the Literature

M_1	M_2	temp. (°C)	solvent	r_1	r_2	r_1 x r_2	ref.
			terminal model				
CHMI[a]	St	60	benzene	0.02	0.11	0.0022	11
PhMI	St	60	benzene	0.01	0.07	0.0007	12
MMI	isobutene	60	chloroform	0.14	0	0	13
MMI	isobutene	60	1,3-dioxane	0.47	0	0	13
2MPhMI[b]	isobutene	60	chloroform	0.26	0	0	14
2MPhMI[b]	isobutene	60	1,3-dioxane	0.47	0	0	14
2MPhMI[b]	isobutene	60	benzene	0.49	0	0	14
4EPhMI[c]	isobutene	60	benzene	2.48	0	0	14
PhMI	diisobutene	60	CH_2ClCH_2Cl	0.16	0.0076	0.0012	8
M_1	M_2	temp. (°C)	solvent	r_{11}	r_{21}	r_{21}/r_{11}	ref.
			penultimate unit model				
EMI[d]	diisobutene	60	CH_2ClCH_2Cl	0.26	1.3	5.0	8
EMI[d]	Lim	60	$PhC(CF_3)_2OH$	0.029	17	590	15
PhMI	Lim	60	$PhC(CF_3)_2OH$	0.0042	19	4500	16
PhMI	Lim	60	chloroform	0.42	6.2	15	17
4MPhMI[e]	Lim	60	chloroform	0.77	35	45	17
PhMI	Lim	60	CH_2Cl_2	0.83	600	720	18
PhMI	β-pinene	60	tetrahydrofuran	0.53	15.4	28	19
PhMI	β-pinene	0	CH_2ClCH_2Cl	0.42	3.4	8.1	19
PhMI	β-pinene	60	CH_2ClCH_2Cl	0.24	10.3	42	19
PhMI	β-pinene	100	CH_2ClCH_2Cl	0.26	51	200	19
PhMI	β-pinene	60	CF_3CH_2OH	0.032	2.5	78	19
PhMI	β-pinene	60	$(CF_3)_3COH$	0.0058	3.2	550	19
PhMI	β-pinene	60	$PhC(CF_3)_2OH$	0.0011	7.6	6900	18

[a] CHMI: *N*-cyclohexylmaleimide. [b] 2MPhMI: *N*-(2-methylphenyl)maleimide. [c] 4EPhMI: *N*-(4-ethylphenyl)-maleimide. [d] EMI: *N*-ethylmaleimide. [e] 4MPhMI: *N*-(4-methylphenyl)maleimide.

7. References

(1) Sadiq, A.; Mahnashi, M. H.; Alyami, B. A.; Alqahtani, Y. S.; Alqarni, A.O.; Rashid, U. Tailoring the Substitution Pattern of Pyrrolidine-2,5-dione for Discovery of New Structural Template for Dual COX/LOX Inhibition. *Bioorg. Chem.*, **2021**, *112*, 104969. [10.1016/j.bioorg.2021.104969]

(2) Yaroslavsky, C.; Bracha, P.; Glaser, R. Preparation, Proton and Carbon-13 Structure Determination, and Fungicidal Activity of Chlorinated 1-Arylamino-1*H*-pyrolle-2,5-diones. *J. Heterocyc. Chem.* **1989**, *26*, 1649-1654. [10.1002/jhet.5570260626]

(3) Rubinstein, H.; Skarbek, J. E. Reactions of 3-Carboxyacryloylhydrazines and the Formation of Maleimides, Isomaleimides, and Pyridazinones. *J. Org. Chem.* **1971**, *36*, 3372-3376. [10.1021/jo00821a023]

(4) Cheng, S.; Comer, D. D. An Alumina-Catalyzed Michael Addition of Mercaptans to *N*-Anilinomaleimides and its Application to the Solution-Phase Parallel Synthesis of Libraries. *Tetrahedron Lett.* **2002**, *43*, 1179-1181. [10.1016/S0040-4039(01)02401-7]

(5) Hiran, B. L.; Choudhary, J.; Paliwal, S. N.; Choudhary, P. R. Homo and Copolymerization of *N*-(Phenylamino)maleimide: Synthesis and Characterization. *J. Indian Chern. Soc*. **2007**, *84*, 1153-1158.

(6) Conley, N. R.; Hung, R. J.; Willson, C. G. A New Synthetic Route to Authentic *N*-Substituted Aminomaleimides. *J. Org. Chem.* **2005**, *70*, 4553-4555. [10.1021/jo048031q]

(7) Hisano, M.; Takeda, K.; Takashima, T.; Jin, Z.; Shiibashi, A.; Matsumoto, A. Sequence Controlled Radical Polymerization of *N*-Substituted Maleimides with 1-Methylenebenzocycloalkanes and the Characterization of the Obtained Copolymers with Excellent Thermal Resistance and Transparency. *Macromolecules* **2013**, *46*, 3314-3323. [DOI:10.1021/ma400454u]

(8) Hisano, M.; Takeda, K.; Takashima, T.; Jin, Z.; Shiibashi, A.; Matsumoto, A. Sequence Controlled Radical Copolymerization of *N*-Substituted Maleimides with Olefins and Polyisobutene Macromonomers To Fabricate Thermally Stable and Transparent Maleimide Copolymers with Tunable Glass Transition Temperatures and Viscoelastic Properties. *Macromolecules* **2013**, 46, 7733-7744. [**DOI:**10.1021/ma401499v]

(9) Wongwalikhit, K.; Suwabunnakeeree, R.; Kihara, N. Synthesis of Oxidative Degradation of Lucine-Based Poly(diacylhydrazine). *Polymers* **2024**, *16*, 1222. [10.3390/polym16091222]

(10) Shimohiro, T.; Katayama, M.; Kihara, N. Oxidative Degradation of Acylsemicarbazide and Its Polymers. *Chem. Lett.* **2025**, *54*, upaf209. [10.1093/chemle/upaf209]

(11) Otsu, T.; Matsumoto, A.; Kubota, T. Increase in Thermal Stability of Vinyl Polymers through Radical Copolymerization with *N*-Cyclohexylmaleimide. *Polym. Inter.* **1991**, *25*, 179-184. [10.1002/pi.4990250309]

(12) Matsumoto, A.; Kubota, T.; Otsu, T. Radical Polymerization of *N*-(Alkyl-Substituted phenyl)-maleimides: Synthesis of Thermally Stable Polymers Soluble in Nonpolar Solvents. *Macromolecules* **1990**, *23*, 4508-4513. [10.1021/ma00223a002]

(13) Doi, T.; Akimoto, A.; Matsumoto, A.; Otsu, T. Radical Copolymerization of *N*-Alkylmaleimides with Isobutene and the Properties of the Resulting Alternating Copolymers. *J. Polym. Sci., Part A: Polym. Chem.* **1996**, *34*, 367-373. [10.1002/(SICI)1099-0518(199602)34:3<367::AID-POLA5>3.0.CO;2-R]

(14) Doi, T.; Akimoto, A.; Matsumoto, A.; Oki, Y.; Otsu, T. Alternating Copolymerization of N-(Alkyl-substituted phenyl)maleimides with Isobutene and Thermal Properties of the Resulting Copolymers. *J. Polym. Sci., Part A: Polym. Chem.* **1996**, *34*, 2499-2505. [10.1002/(SICI)1099-0518(19960915)34:12<2499::AID-POLA23>3.0.CO;2-2]

(15) Matsuda, M.; Satoh, K.; Kamigaito, M. 1:2-Sequence-Regulated Radical Copolymerization of Naturally Occurring Terpenes with Maleimide Derivatives in Fluorinated Alcohol. *J. Polym. Sci., Part A: Polym. Chem.* **2013**, *51*, 1774-1785. [DOI:10.1002/pola.26556]

(16) Satoh, K.; Matsuda, M.; Nagai, K.; Kamigaito, M. AAB-Sequence Living Radical Chain Copolymerization of Naturally Occurring Limonene with Maleimide: An End-to-End Sequence-Regulated Copolymer**.** *J. Am. Chem. Soc.* **2010**, *132*, 10003-10005. [10.1021/ja1042353]

(17) Terada, S.; Matsumoto, A. Role of *N*-Substituents of Maleimides on Penultimate Unit Effect for Sequence Control during Radical Copolymerization. *Polym. J.* **2019**, *51*, 1137-1146. [DOI:10.1038/s41428-019-0227-y]

(18) Matsuda, M.; Satoh, K.; Kamigaito, M. Periodically Functionalized and Grafted Copolymers via 1:2-Sequence-Regulated Radical Copolymerization of Naturally Occurring Functional Limonene and Maleimide Derivatives. *Macromolecules* **2013**, *46*, 5473-5482. [DOI: 10.1021/ma401021d]

(19) Yamamoto, D.; Matsumoto, A. Penultimate Unit and Solvent Effects on 2:1 Sequence Control during Radical Copolymerization of *N*-Phenylmaleimide with β-Pinene. *Macromol. Chem. Phys.* **2012**, *213*, 2479-2485. [10.1002/macp.201200421]
